# Epigenetic priming of neural progenitors by Notch enhances Sonic hedgehog signaling and establishes gliogenic competence

**DOI:** 10.1101/gad.352555.124

**Published:** 2025-07-01

**Authors:** Luuli N. Tran, Ashwini Shinde, Kristen H. Schuster, Aiman Sabaawy, Emily Dale, Madalynn J. Welch, Trevor J. Isner, Sylvia A. Nunez, Fernando García-Moreno, Charles G. Sagerström, Bruce H. Appel, Santos J. Franco

**Affiliations:** 1 Molecular Biology Graduate Program, University of Colorado Anschutz Medical Campus, Aurora, Colorado 80045, USA;; 2 Neuroscience Graduate Program, University of Colorado Anschutz Medical Campus, Aurora, Colorado 80045, USA;; 3 Gates Summer Internship Program, Gates Institute, University of Colorado Anschutz Medical Campus, Aurora, Colorado 80045, USA;; 4 Department of Pediatrics, Section of Developmental Biology, University of Colorado Anschutz Medical Campus, Aurora, Colorado 80045, USA;; 5 Cell Biology, Stem Cells, and Development Graduate Program, University of Colorado Anschutz Medical Campus, Aurora, Colorado 80045, USA;; 6 Achucarro Basque Center for Neuroscience, Edificio Sede del Parque Científico de la Universidad del País Vasco/Euskal Herriko Unibertsitatea, Leioa 48940, Spain;; 7 Program in Pediatric Stem Cell Biology, Children's Hospital Colorado, Aurora, Colorado 80045, USA

**Keywords:** epigenetic priming, Notch, Sonic hedgehog, cell fate, competence, gliogenesis, oligodendrocyte, forebrain, cortex

## Abstract

The remarkable cell diversity of multicellular organisms relies on the ability of multipotent progenitor cells to generate distinct cell types at the right times and locations during embryogenesis. A key question is how progenitors establish competence to respond to the different environmental signals required to produce specific cell types at critical developmental time points. We addressed this in the mouse developing forebrain, where neural progenitor cells must switch from producing neurons to making oligodendrocytes in response to increased Sonic hedgehog (SHH) signaling during late embryogenesis. We show that progenitor responses to SHH are regulated by Notch signaling, thus permitting proper timing of the neuron–oligodendrocyte switch. Notch activity epigenetically primes genes associated with the oligodendrocyte lineage and SHH pathway, enabling amplified transcriptional responses to endogenous SHH and robust oligodendrogenesis. These results reveal a critical role for Notch in facilitating progenitor competence states and influencing cell fate transitions at the epigenetic level.

## Abstract (precis)

In this study, Tran et al. show that during neural progenitor differentiation in the forebrain, Notch signaling controls the switch from neuronal fates to oligodendrocyte fates in response to Sonic hedgehog signaling during late embryogenesis. Notch epigenetically primes and amplifies gliogenic transcriptional programs, increasing oligodendrogenesis and facilitating progenitor competence and proper developmental timing.

Cell fate specification is a critical process by which multipotent progenitor cells acquire distinct identities with specialized functions. To achieve this, progenitors must respond appropriately to environmental signals to generate the right cell types at the proper times during embryogenesis. A prime example of this process is found in the developing central nervous system (CNS), where multiple different types of neurons and glial cells originate from a shared pool of neural progenitor cells in a spatially and temporally regulated manner. In the developing dorsal forebrain, neural progenitors first generate excitatory neurons before they start producing glial cells like astrocytes and oligodendrocytes in a process known as the “neuron–glia switch” (Sauvageot 2002; Guillemot 2007; Franco and Müller 2013). This switch provides an excellent model to investigate the mechanisms that govern the acquisition of diverse fates from a common pool of neural progenitors.

Proper timing of the neuron–glia switch relies on precisely coordinated interactions between cell-intrinsic factors, such as epigenetic state and gene expression profile, and extracellular signals that instruct progenitors to commence a gliogenic transcriptional program (Sauvageot 2002; Guillemot 2007; Franco and Müller 2013). For example, during early embryonic development, the morphogen signaling molecule Sonic hedgehog (SHH) is highly expressed in the ventral forebrain to pattern the dorsal–ventral axis, allowing for neurogenesis to occur in dorsal regions (Fuccillo et al. 2004). Studies from our laboratory showed that SHH signaling increases in the dorsal forebrain during later stages of embryonic development, initiating the transition from neurogenesis to the formation of oligodendrocyte lineage cells (Winkler et al. 2018). Importantly, we found that the beginning of oligodendrogenesis overlaps with the end of neurogenesis, so that only some progenitors form oligodendrocytes in response to the SHH ligand, while others continue to produce neurons. This suggests that additional pathways are required to make progenitors competent to produce oligodendrocyte lineage cells upon SHH exposure.

Multiple studies have demonstrated that the intrinsic properties of neural progenitors change over developmental time, and thus their competence to respond to specific extracellular signals also changes (Kohwi and Doe 2013; Yuzwa et al. 2017; Li et al. 2020; Katada et al. 2021). Although neural progenitors maintain a core cellular identity, single-cell transcriptomics studies suggest that progenitors are transcriptionally primed with a mixed cell identity before a specific neuronal subtype lineage is resolved (Yuzwa et al. 2017; Li et al. 2020). Additionally, the chromatin landscape provides another key regulatory step in imposing divergent cell fates. For instance, temporal changes in chromatin dynamics of neural progenitors determine whether they generate neurons or astrocytes in response to BMP signaling (Katada et al. 2021). These studies point toward the intriguing possibility that neural progenitors may require a specific intrinsic molecular state that is competent to produce oligodendrocyte lineage cells in response to SHH signaling.

We previously demonstrated that the Notch signaling pathway both promotes a progenitor state and is required for the proper timing and scale of dorsal forebrain oligodendrogenesis (Tran et al. 2023). Here, we show that Notch signaling is required for SHH-mediated oligodendrogenesis, providing us with a framework to interrogate the intrinsic progenitor state that is competent to respond to SHH and to specify the oligodendrocyte lineage. We used Notch gain-of-function mutants to profile the transcriptomes and chromatin landscapes of neural progenitors experiencing Notch pathway overactivation during the neuron–glia switch in the dorsal forebrain. We found that Notch activation reorganized the genome in favor of a more gliogenic and less neurogenic chromatin state and promoted accessibility of chromatin near SHH pathway genes. Interestingly, at the transcriptional level, higher Notch signaling initially promoted an undifferentiated progenitor identity that lacked expression of mRNAs known to drive cell fate specification and differentiation. However, a day later during the peak of the neuron–glia switch, Notch activation increased oligodendrogenesis. Finally, mRNA sequencing revealed upregulation of SHH pathway components, and in vivo reporter assays revealed that Notch activation amplified SHH transcriptional output. These data illustrate a model in which Notch signaling maintains an undifferentiated epigenetic and transcriptomic state in neural progenitors while also establishing a chromatin landscape that inhibits neurogenesis and primes progenitors for a gliogenic program. Later, primed progenitors experience an enhanced response to SHH signaling and initiate robust oligodendrocyte production, thus contributing to the proper timing and scale of the neuron–glia switch.

## Results

### Notch signaling regulates the production of oligodendrocyte lineage cells in response to SHH

**Figure 1.**
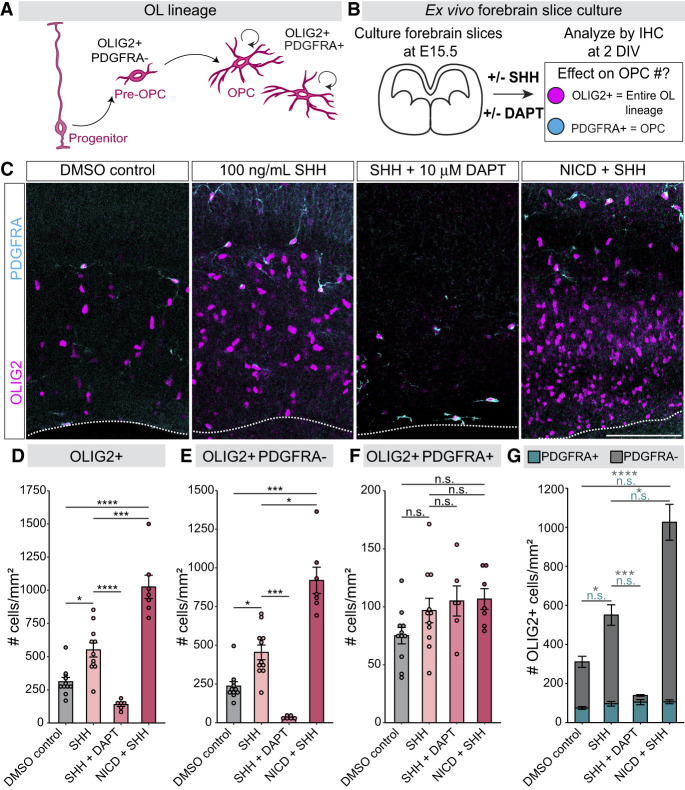
Notch pathway inhibition blocks SHH-induced oligodendrogenesis in forebrain slice cultures. (*A*) Schematic of OL lineage progression. Pre-OPCs derived from dorsal forebrain progenitors express OLIG2 and transition into OPCs once they start expressing PDGFRA. OPCs proliferate and populate the dorsal pallium. (OL) Oligodendrocyte, (OPC) oligodendrocyte precursor cell. (*B*) Schematic of the forebrain slice culture approach. Brains of E15.5 wild-type or NICD mouse embryos were sectioned and cultured with or without DAPT and SHH. At 2 DIV, forebrain slices were fixed and stained for OLIG2 and PDGFRA to identify oligodendrocyte lineage cells. (DIV) Days in vitro, (IHC) immunohistochemistry. (*C*) Representative images of OLIG2^+^ and OLIG2^+^ PDGFRA^+^ oligodendrocyte lineage cells in the dorsal pallium of wild-type forebrain slices cultured in DMSO control, 100 ng/mL SHH, and SHH + DAPT, as well as NICD forebrain slices cultured in 100 ng/mL SHH. Dotted lines indicate the ventral (bottom) limits of the pallium. Scale bar, 100 µm. (*D*–*G*) Quantification of oligodendrocyte lineage cells from cultured slices. Total numbers of OLIG2^+^ cells (*D*), OLIG2^+^ PDGFRA^−^ cells (*E*), and OLIG2^+^ PDGFRA^+^ cells (*F*) were counted per square millimeter in the pallium of DMSO control, SHH-treated, SHH + DAPT-treated, and NICD + SHH-treated forebrain slice cultures. Graphs show the average ± SEM among biological replicates. Data from *E* and *F* were regraphed in *G* to show the proportion of total OLIG2^+^ cells that are either PDGFRA^+^ or PDGFRA^−^. One-way ANOVA (with Tukey's post hoc) comparing DMSO, SHH, and NICD + SHH: *P* = 2.01 × 10^−08^ (*D*) and *P* = 0.15, n.s. (*E*,*G*). (*D*) Welch ANOVA (with Games–Howell post hoc) comparing DMSO, SHH, and SHH + DAPT: *P* = 1.68 × 10^−06^. (*E*,*G*) One-way ANOVA comparing DMSO, SHH, and SHH + DAPT: *P* = 0.11, n.s. (*F*,*G*) Kruskal–Wallis (with Dunn's) comparing DMSO, SHH, and NICD + SHH: *P* = 3.07 × 10^−05^. (*F*,*G*) Kruskal–Wallis (with Dunn's) comparing DMSO, SHH, and SHH + DAPT: *P* = 5.23 × 10^−05^. (*) *P* < 0.05, (***) *P* < 0.0001, (****) *P* < 0.00001, (ns) not significant. *N* = 11 DMSO control, *N* = 11 SHH, *N* = 6 SHH + DAPT, *N* = 7 SHH + NICD. See also Supplemental Figure S1.

We previously showed that the SHH and Notch signaling pathways are individually critical for proper oligodendrogenesis from dorsal forebrain progenitors during the neuron–glia switch (Winkler et al. 2018; Tran et al. 2023). Given that Notch signaling can impact SHH signaling in other developmental contexts (Kong et al. 2015; Stasiulewicz et al. 2015; Ringuette et al. 2016; Ravanelli et al. 2018; Jacobs and Huang 2019, 2021), we hypothesized that Notch pathway activation controls oligodendrogenesis in part by regulating progenitor responses to SHH. To test this hypothesis, we first asked whether Notch signaling is required for SHH-induced oligodendrogenesis using an ex vivo forebrain slice culture system (Fig. 1). Dorsal forebrain progenitors begin generating the oligodendrocyte lineage during late embryogenesis, starting with the production of OLIG2^+^ glial progenitor cells, some of which then express PDGFRA as they transition to oligodendrocyte precursor cells (OPCs) (Fig. 1A; Pringle and Richardson 1993; Winkler et al. 2018). We cut forebrain slices from mouse embryos right before the neuron–glia switch at embryonic day (E) 15.5 and cultured them for 2 days to analyze oligodendrogenesis by staining for OLIG2 and PDGFRA (Fig. 1B). Following Notch receptor activation, γ-secretases cleave the Notch intracellular domain (NICD), which forms a transcriptional complex with the cofactor RBPJ to activate target gene expression (Andersson et al. 2011). We blocked Notch signaling in slice cultures by adding the γ-secretase inhibitor DAPT and stimulated the SHH pathway by adding recombinant SHH ligand (Fig. 1B). We previously showed that DAPT treatment severely reduces the number of OLIG2^+^ cells in the pallium (Tran et al. 2023). In contrast, we found here that SHH treatment significantly increased OLIG2^+^ cells by twofold, consistent with its role in promoting dorsal oligodendrogenesis. However, this SHH-induced increase in OLIG2^+^ cells was completely blocked when slices were treated with both SHH and DAPT together (Fig. 1C,D). Conversely, we found the opposite when Notch signaling was activated. To activate the Notch pathway specifically in the dorsal lineage, we crossed *Emx1-Cre* mice (Gorski et al. 2002) to the *R26-Lox-Stop-Lox-NICD* line (Murtaugh et al. 2003). The resulting mice, referred to here as NICD, overexpress NICD in *Emx1*^+^ dorsal forebrain progenitors (Han et al. 2023). We found that adding SHH to slices from NICD brains resulted in an even greater increase in OLIG2^+^ cells compared with SHH alone (Fig. 1C,D). Together, these data demonstrate that SHH-mediated oligodendrogenesis in the dorsal forebrain can be modulated by Notch signaling.

Most OLIG2^+^ cells in the pallium at this stage are pre-OPCs that are newly derived from dorsal progenitors and do not express PDGFRA yet. Conversely, we previously showed that the majority of OLIG2^+^ PDGFRA^+^ cells in the E15.5 pallium are earlier-born OPCs that derive from the ventral forebrain (Kessaris et al. 2006; Winkler et al. 2018) and that their numbers are unchanged following DAPT treatment (Tran et al. 2023). Similarly, we found here that SHH, SHH + DAPT, and SHH + NICD treatments did not significantly affect OLIG2^+^ PDGFRA^+^ OPC numbers in E15.5 slice cultures (Fig. 1C,F,G). This likely reflects the earlier birthdate of ventrally derived OPCs (Kessaris et al. 2006) and their maturation beyond the point of requiring Notch signaling (Tran et al. 2023). On the other hand, SHH treatment increased OLIG2^+^ PDGFRA^−^ cells by nearly twofold, and SHH + DAPT treatment again blocked this effect (Fig. 1C,E). We compared the proportion of OLIG2^+^ cells that are PDGFRA^+^ versus PDGFRA^−^ and observed that the OLIG2^+^ PDGFRA^−^ population is the most strongly affected by SHH and DAPT treatments (Fig. 1G). Together with our prior studies (Winkler et al. 2018; Tran et al. 2023), these results suggest a role for Notch and SHH pathway cross-talk in regulating the initial specification and generation of oligodendrocyte lineage cells from dorsal progenitors.

Neural progenitor responses to SHH signaling depend on the concentration of SHH ligand and duration of exposure (Briscoe and Ericson 2001; Dessaud et al. 2007). Previous studies in the zebrafish, chick, and mouse spinal cord demonstrated that progenitor responses to a given SHH concentration are potentiated by increased Notch signaling and are decreased when Notch signaling is blocked (Kong et al. 2015; Ravanelli et al. 2018). This requirement of Notch signaling for optimal SHH-dependent cellular responses can be overcome by increasing SHH signaling directly through its downstream target, Smoothened (SMO) (Kong et al. 2015). We therefore wondered whether increasing SHH signaling through SMO activation could promote oligodendrogenesis during Notch inhibition in the dorsal forebrain. We tested this in vivo using in utero electroporation of dorsal forebrain progenitors (Supplemental Fig. S1A). To block Notch signaling, we electroporated dominant-negative (DN) RBPJ (DN-RBPJ-IRES-mtagBFP2), which inhibits Notch signaling through disruption of the DNA-binding domain of RBPJ (Chung et al. 1994). We also generated a plasmid that expresses a constitutively active version of SMO (SMOA1-IRES-GFP), which overactivates the SHH pathway cell-autonomously (Taipale et al. 2000; Dey et al. 2012). We coelectroporated E15.5 mouse embryonic brains with SMOA1 combined with DN-RBPJ or with BFP-only control and analyzed brains at E18.5 by immunohistochemistry for oligodendrocyte lineage markers (Supplemental Fig. S1A). As in our prior studies (Tran et al. 2023), DN-RBPJ decreased the proportion of electroporated cells that were OLIG2^+^ and OLIG2^+^ PDGFRA^+^ (Supplemental Fig. S1B–D). Conversely, SMOA1 increased the proportion of OLIG2^+^ cells by almost fourfold (Supplemental Fig. S1B,C) and that of OLIG2^+^ PDGFRA^+^ OPCs by threefold (Supplemental Fig. S1B,D). Coelectroporation with SMOA1 and DN-RBPJ together increased OLIG2^+^ and OLIG2^+^ PDGFRA^+^ electroporated cells at proportions similar to that of SMOA1 alone (Supplemental Fig. S1B–D). These results indicate that the requirement of Notch signaling for progenitors to produce an optimal SHH response during the neuron-to-glia switch (E15.5–E18.5) can be bypassed by increasing SHH signaling. Altogether, our data and previous studies demonstrate that Notch signaling can potentiate cellular responses to SHH signaling.

### Notch activation in progenitors promotes an undifferentiated transcriptomic identity

**Figure 2.**
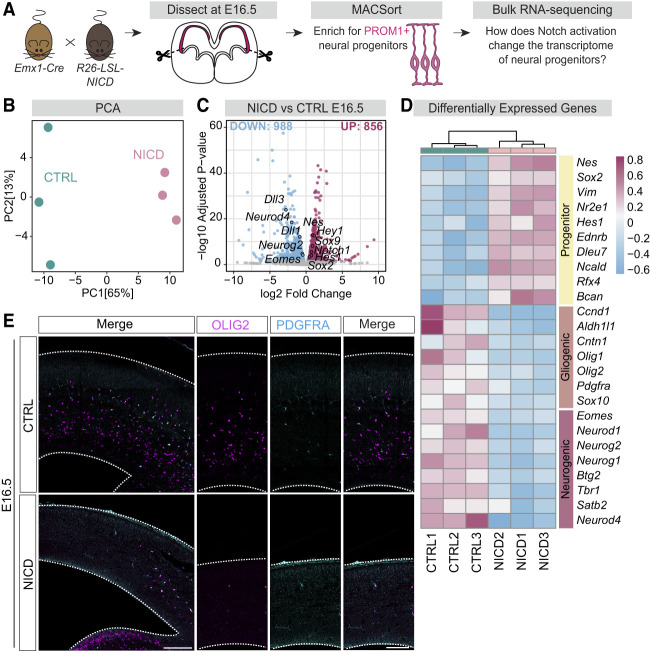
RNA-seq analysis of NICD dorsal progenitors reveals an undifferentiated, stem-like transcriptome. (*A*) Schematic of bulk RNA-seq workflow. *Emx1-Cre* mice were crossed to *R26-LSL-NICD* mice to activate Notch signaling in *Emx1*^+^ dorsal progenitors and their progeny. The dorsal forebrains of CTRL and NICD mouse embryos were dissected at E16.5, dissociated, and MACSorted to enrich for PROM1^+^ dorsal progenitors for bulk RNA-seq. (*B*) PCA plot indicating the separation of CTRL (*N* = 3) and NICD (*N* = 3) transcriptomes by condition. (PCA) Principal component analysis. (*C*) Volcano plot representing differentially expressed genes between CTRL and NICD. Differentially expressed genes with adjusted *P*-value < 0.05 were considered to be significant. Nine-hundred-eighty-eight genes were significantly downregulated (blue) and 856 genes were upregulated (pink) in NICD compared with CTRL. The *Y*-axis represents the −log_10_-adjusted *P*-value, and the *X*-axis represents the log_2_ fold change between CTRL and NICD. (*D*) Heat map representing selected differentially expressed genes related to progenitor identity (*top*), gliogenic identity (*middle*), and neurogenic identity (*bottom*). The heat map scale shows variance-stabilized gene expression levels, with colors representing relative expression levels across samples. (*E*) Representative images of CTRL and NICD E16.5 forebrains, outlined in white and stained for OLIG2 and PDGFRA. (*Left*) Overview images. Scale bar, 200 µm. (*Right*) Zoomed-in images. Dotted lines indicate the dorsal (*top*) and ventral (*bottom*) limits of the pallium. Scale bar, 100 µm. See also Supplemental Tables S1 and S2.

We next wanted to understand the molecular mechanisms underlying the ability of Notch signaling to enhance neural progenitor responses to SHH signaling. Following Notch pathway activation, the NICD–RBPJ complex can bind to and activate transcription of many target genes, including Notch transcriptional effectors such as *Hes1*. These changes in gene expression may contribute to the ability of progenitors to generate oligodendrocyte lineage cells in response to SHH. To understand Notch's influence on gene expression in neural progenitors, we performed bulk mRNA sequencing (RNA-seq) on dorsal forebrain progenitors experiencing Notch overactivation using the NICD mice. Following dorsal forebrain dissections and dissociations at E16.5, we isolated neural progenitors by magnetic cell sorting (MACS) using an antibody against the progenitor-specific cell surface marker PROM1 (Fig. 2A; Holmberg Olausson et al. 2014). Three biological replicates were obtained and sequenced for NICD and *Emx1-Cre*-only controls (CTRL). Principal component analysis (PCA) of all samples showed separation between CTRL and NICD samples (Fig. 2B), indicating transcriptomes that were different from one another. Differential gene expression analysis (Supplemental Table S1) revealed 856 upregulated genes in progenitors, including known Notch targets like *Hes1, Hey1*, and *Notch1*. In contrast, 988 genes were downregulated by NICD, including Notch ligands that are known to be expressed in cells with low or no Notch signaling, such as *Dll1* and *Dll3* (Fig. 2C). Further analysis of differentially expressed genes between NICD and CTRL revealed a transcriptomic profile that was undifferentiated and progenitor-like. For example, genes related to neurogenesis like *Eomes* and *Neurod4* were downregulated in NICD progenitors, whereas stemness genes like *Sox2* and *Nes* were upregulated (Fig. 2C,D). This result is in line with prior studies demonstrating aberrant neurogenesis and increased progenitor maintenance in the *Emx1-Cre;R26-Lox-Stop-Lox-NICD* dorsal forebrain (Han et al. 2023). Interestingly, genes known to play roles in glial differentiation like *Olig2*, *Pdgfra*, and *Aldh1l1* were also downregulated at this time (Fig. 2D). Transcriptional targets downstream from the SHH pathway were largely unchanged by NICD overexpression (Supplemental Table S2).

We next performed immunohistochemistry (IHC) for markers of the oligodendrocyte lineage on E16.5 NICD and CTRL brains. As reported previously (Han et al. 2023), NICD brains displayed thinner cortices compared with CTRL brains (Fig. 2E). Consistent with the decreased expression of glial differentiation genes in the RNA-seq data, we observed very few OLIG2^+^ or PDGFRA^+^ cells in the pallium of E16.5 NICD mouse embryos (Fig. 2E), suggesting that NICD progenitors had not yet begun producing oligodendrocyte lineage cells. This is in contrast to CTRL brains that had already initiated oligodendrogenesis at E16.5 (Fig. 2E). Overall, RNA-seq analysis and IHC at E16.5 demonstrate that NICD-overexpressing progenitors maintained an undifferentiated identity and did not produce pre-OPCs or OPCs at this time.

### Notch activation changes chromatin accessibility in progenitors

**Figure 3.**
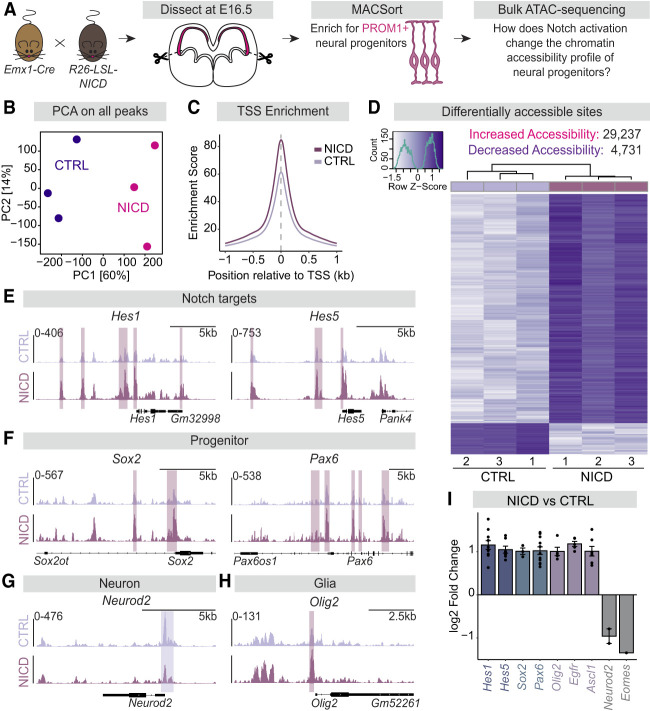
NICD promotes chromatin accessibility in dorsal progenitors as revealed by ATAC-seq. (*A*) Schematic of bulk ATAC-seq workflow. *Emx1-Cre* mice were crossed to *R26-LSL-NICD* mice to activate Notch signaling in *Emx1*^+^ dorsal progenitors and their progeny. The dorsal forebrains of CTRL and NICD mouse embryos were dissected at E16.5, dissociated, and MACSorted to enrich for PROM1^+^ dorsal progenitors for bulk ATAC-seq. (*B*) PCA plot of all accessible chromatin peaks identified in CTRL (*N* = 3) and NICD (*N* = 3) samples. (PCA) Principal component analysis. (*C*) TSS enrichment plot representing normalized read coverage or accessible chromatin around transcription start sites in CTRL and NICD. The *Y*-axis represents an enrichment score or ratio of read density at the TSS compared with background read density. The *X*-axis represents the position in kilobases relative to the TSS. (TSS) Transcription start site. (*D*) Heat map representing the *Z*-scores of normalized read counts for all differentially accessible sites that either increased or decreased in NICD compared with CTRL. Peaks are considered significant with an adjusted *P*-value < 0.05. The *X*-axis shown in the scale reflects the row *Z*-scores, which are based on the standardized deviation of accessibility from the mean, and the *Y*-axis represents the count of peaks. (*E*–*H*) Genome tracks of peaks surrounding genes related to the Notch pathway (*E*), progenitor identity (*F*), neuron identity (*G*), and glia identity (*H*). Peaks highlighted in pink are significant peaks assigned to the shown genes and enriched in NICD, while peaks highlighted in purple are significant peaks enriched in CTRL. Scales shown on the *Y*-axis represent signal density across the genome. (*I*) Graph representing the log_2_ fold change of all significant peaks associated with the list of selected genes, with each dot representing a peak. Peaks were assigned to genes based on distance to the nearest TSS. See also Supplemental Table S3.

Because NICD dorsal progenitors appeared more progenitor-like and undifferentiated based on transcriptome analyses, we wondered whether Notch's influence on progenitor responsiveness to SHH and ability to generate glia may instead be at the level of chromatin accessibility. The chromatin landscape plays key roles in regulating gene expression and thus determining the cell identity of progenitors. To test whether Notch signaling can influence the chromatin landscape in progenitors, we used assay for transposase-accessible chromatin sequencing (ATAC-seq) to profile accessible chromatin regions in sorted PROM1^+^ dorsal progenitors (Fig. 3A). Three biological replicates were obtained for NICD and CTRL dorsal forebrain progenitors at E16.5 and sequenced for accessible chromatin. Similar to our RNA-seq results, NICD and CTRL dorsal progenitors had different chromatin accessibility profiles based on PCA (Fig. 3B). In particular, open transcription start sites (TSSs) were more enriched in NICD than in CTRL (Fig. 3C). We further analyzed genome-wide differences in chromatin accessibility (Supplemental Table S3) and identified 29,237 sites that increased in accessibility in NICD dorsal progenitors and 4731 sites that decreased in accessibility in NICD compared with CTRL progenitors (Fig. 3D). Because increased chromatin accessibility is associated with a multipotent progenitor state (Stergachis et al. 2013), our ATAC-seq results are in line with our RNA-seq results suggesting that NICD promotes an undifferentiated progenitor identity.

**Figure 4.**
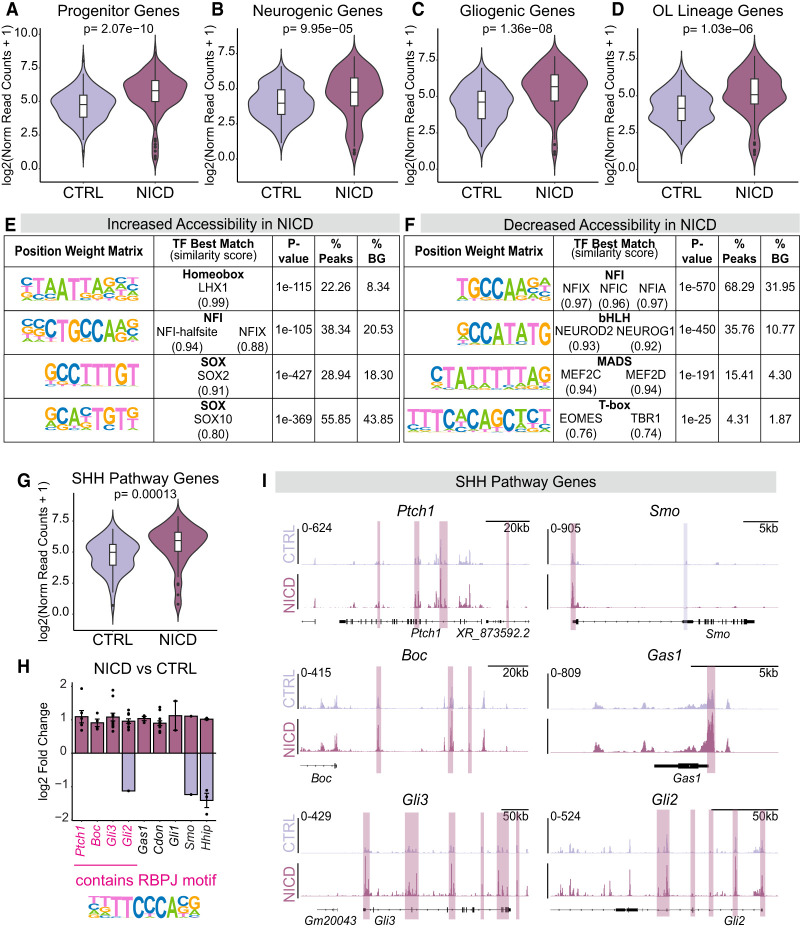
Notch overactivation reorganizes chromatin surrounding cell identity genes and SHH pathway genes. (*A*–*D*) Plots representing the normalized read counts or accessibility of significant peaks assigned to progenitor genes (*A*), neurogenic genes (*B*), gliogenic genes (*C*), and OL lineage genes (*D*). Wilcoxon rank sum test was used to determine significance. (OL) Oligodendrocyte lineage. (*E*,*F*) Transcription factor binding motifs identified by HOMER de novo motif enrichment analysis of the 29,237 increased accessibility sites (*E*) and 4731 decreased accessibility sites (*F*) in NICD. (% BG) Percentage of background sequences. (*G*) Normalized read count plot of significant peaks assigned to genes related to the SHH signaling pathway. (*H*) Graph representing the log_2_ fold change of all significant peaks associated with selected SHH pathway genes, with each dot representing a peak. Peaks were assigned to genes based on distance to the nearest TSS. (TSS) Transcription start site. Gene names colored in pink have peaks that contain the RBPJ binding motif shown *below*. (*I*) Genome tracks showing chromatin accessibility peaks surrounding genes related to the SHH pathway. Peaks highlighted in pink are significant peaks assigned to the shown genes and enriched in NICD, while peaks highlighted in purple are significant peaks enriched in CTRL. See also Supplemental Tables S4–S6.

To further understand the influence of NICD on cell identity, we analyzed genes associated with differentially accessible regions. Notch target genes like *Hes1* and *Hes5* exhibited chromatin peaks that were significantly more accessible in NICD compared with CTRL (Fig. 3E,I). Similarly, progenitor identity genes, like *Sox2* and *Pax6*, also showed increased accessibility in NICD progenitors (Fig. 3F,I). NICD progenitors had decreased chromatin accessibility peaks near certain neurogenic genes like *Neurod2* and *Eomes* (Fig. 3G,I), consistent with the RNA-seq results indicating a less neurogenic state. Interestingly, however, peaks near genes associated with gliogenesis like *Olig2, Egfr*, and *Ascl1* (Fig. 3H,I) exhibited increased accessibility. These results indicate that Notch activation with NICD reorganizes chromatin near lineage specification genes. We therefore wanted to better understand the overall influence of NICD on the chromatin landscape surrounding broader categories of genes associated with different cell fates. To do so, we curated gene lists known to be important for progenitor, neurogenic, and gliogenic identities and compared the normalized cumulative accessibility of chromatin near the genes in each identity class. Interestingly, NICD-overexpressing progenitors exhibited abundant accessibility near all three gene categories (Fig. 4A–C), as well as genes specific to the oligodendrocyte lineage (Fig. 4D). Therefore, whereas the transcriptomic profile of NICD progenitors indicated an undifferentiated and progenitor-like state, their chromatin accessibility profile suggested that they had increased accessibility nearby both neurogenic and gliogenic genes that were not yet expressed. Together with our RNA-seq data, our ATAC-seq analyses point toward the possibility that these genes with increased accessibility are poised to be expressed but require specific factors that are not yet present. Such factors may be transcriptional regulators that play key roles in determining cellular identity.

Therefore, we next asked which transcription factor motifs were present in both more accessible and less accessible sites in NICD progenitors. De novo motif enrichment analysis of more accessible sites predicted motifs of transcription factors like the LHX family, the NFI family, and SOX10 (Fig. 4E), all of which have known roles in gliogenesis in the forebrain (Stolt et al. 2002; Cebolla and Vallejo 2006; Symmank et al. 2019; Welle et al. 2021). The motif that matches SOX2, which influences progenitor identity (Bylund et al. 2003; Graham et al. 2003), was also identified (Fig. 4E). In contrast, the motifs identified in less accessible sites (i.e., enriched in CTRL) matched with transcription factors like NEUROD2, MEF2C, and EOMES (Fig. 4F), which are known regulators of neuron identity (Olson et al. 2001; Arnold et al. 2008; Li et al. 2008; Hevner 2019). Furthermore, these motifs were identified near genes associated with neuron differentiation and maturation (Supplemental Table S4). Because these motifs were found in sites that are less accessible in NICD-overexpressing progenitors, this suggests that NICD may delay neuron differentiation and maturation by decreasing chromatin accessibility to neurogenic transcription factors. Indeed, NICD expression does not completely block neuron production but greatly altered its timing and efficiency (Han et al. 2023). On the other hand, LHX, NFI, and SOX10 motifs were enriched in regions with increased accessibility in NICD progenitors and were found near genes important for gliogenesis (Supplemental Table S5). Interestingly, motifs matching the binding motifs of the NFI family of transcription factors were identified in both more and less accessible sites, consistent with their known roles in both neurogenesis and gliogenesis (Mason et al. 2009). Altogether, our ATAC-seq and RNA-seq data from E16.5 dorsal forebrain progenitors indicate that Notch signaling reinforces progenitor stemness at the genomic and transcriptional levels while also epigenetically priming progenitors to turn on gliogenic genes that are not yet expressed.

### Notch activation promotes chromatin accessibility near SHH pathway component genes

Because Notch signaling is required for SHH-induced oligodendrogenesis and NICD promoted chromatin accessibility near oligodendrocyte fate specification genes, we next asked whether Notch activation can regulate the chromatin landscape surrounding genes known to promote SHH signaling. We compared the normalized accessibility of peaks associated with genes that positively regulate SHH pathway activity between CTRL and NICD. We found that NICD had increased accessibility near SHH pathway genes (Fig. 4G). When we compared the log_2_ fold change of accessibility at all peaks that were associated with SHH pathway component genes, we found that almost all chromatin accessibility changes were in the positive direction (Fig. 4H,I). Although the RBPJ motif was not identified in the de novo motif enrichment analysis, a search for instances of the RBPJ motif in our ATAC-seq data revealed that several differential chromatin peaks contained RBPJ motifs (Supplemental Table S6), including gliogenic genes such as *Ascl1*, *Egfr*, and *Olig2*, suggesting that NICD–RBPJ may regulate some of these genes directly. RBPJ motifs were also identified within differentially accessible regions associated with SHH pathway component genes (Fig. 4H, colored in pink). Altogether, these ATAC-seq analyses suggest that NICD promotes chromatin accessibility near gliogenic and SHH pathway component genes.

### Notch signaling primes progenitors for enhanced SHH signaling and robust oligodendrogenesis

**Figure 5.**
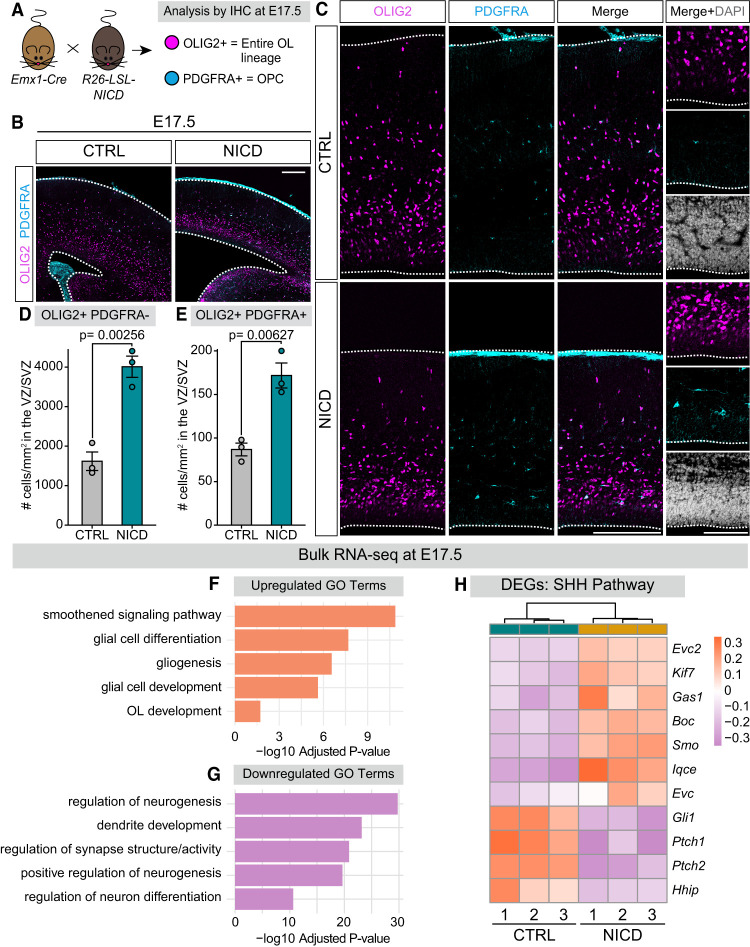
NICD brains have increased gliogenesis and upregulated expression of SHH pathway components at E17.5. (*A*) Schematic of workflow and IHC. *Emx1-Cre* mice were crossed to *R26-LSL-NICD* mice to activate Notch signaling in *Emx1*^+^ dorsal progenitors and their progeny. Brains were dissected at E17.5 and stained for OL markers. (OL) Oligodendrocyte, (OPC) oligodendrocyte precursor cell, (IHC) immunohistochemistry. (*B*) Representative overview images of CTRL and NICD E17.5 forebrains, outlined in white, and stained for OLIG2 and PDGFRA. Scale bar, 200 µm. (*C*, *left*) Representative images of CTRL and NICD dorsal palliums. Dotted lines indicate dorsal (*top*) and ventral (*bottom*) limits. Scale bar, 100 µm. Zoomed-in images at the far *right* show the VZ/SVZ, where dorsal progenitors reside. Scale bar, 100 µm. (*D*,*E*) Quantification of the number of OLIG2^+^ PDGFRA^−^ (*D*) and OLIG2^+^ PDGFRA^+^ (*E*) cells per square millimeter within the VZ/SVZ, ±SEM. (VZ/SVZ) Ventricular zone/subventricular zone. (*F*,*G*) GO enrichment analysis on upregulated (*F*) and downregulated (*G*) genes identified by RNA-seq. The *X*-axis represents the −log_10_-adjusted *P*-value, and GO terms are on the *Y*-axis. (GO) Gene ontology. (*H*) Heat map representing selected differentially expressed genes related to the SHH pathway. The heat map scale shows variance-stabilized gene expression levels, with colors representing relative expression levels across samples. (DEG) Differentially expressed genes. See also Supplemental Figures S2 and S3 and Supplemental Table S7.

Our results indicate that Notch signaling may be priming neural progenitors epigenetically for enhanced SHH signaling and gliogenesis while also transcriptionally repressing glial and neuronal differentiation. This is consistent with our prior studies demonstrating that Notch signaling plays dual roles in setting the timing of the neuron–glia switch by both positively and negatively regulating oligodendrogenesis (Tran et al. 2023). We therefore hypothesized that NICD-overexpressing progenitors that are epigenetically primed but transcriptionally repressed at E16.5 would exhibit enhanced SHH signaling and robust oligodendrogenesis at E17.5, which is the peak of the SHH-dependent neuron–glia transition (Winkler et al. 2018). To test this hypothesis, we first stained sections from NICD and CTRL brains with oligodendrocyte lineage markers at E17.5 (Fig. 5A). In contrast to the lack of OLIG2^+^ and OLIG2^+^ PDGFRA^+^ cells in NICD brains at E16.5 (Fig. 2E), we observed robust oligodendrogenesis in NICD-overexpressing brains at E17.5 (Fig. 5B,C). We quantified the density of OLIG2^+^ and OLIG2^+^ PDGFRA^+^ cells in the ventricular and subventricular zones (VZ/SVZ), where they are first specified and generated, and found a significant increase in NICD-overexpressing brains compared with CTRL brains (Fig. 5D,E). These data suggest that increased Notch signaling in NICD brains delayed oligodendrogenesis at E16.5 but enhanced oligodendrocyte lineage output 1 day later at E17.5.

The increased gliogenesis observed at E17.5 could be a result of multiple impacts of Notch signaling on neural progenitors. First, the NICD mice have a larger progenitor pool resulting from delayed neuronal differentiation (Han et al. 2023), which could allow for increased glial cell production as these progenitors start to differentiate in response to SHH signaling. In addition, increased Notch signaling in these progenitors may also make them more likely to undergo gliogenesis when they finally differentiate. To test this second possibility, we repeated our bulk RNA-seq experiments of PROM1^+^ isolated neural progenitors, this time from E17.5 NICD and CTRL brains. NICD and CTRL samples clearly separated in our PCA, indicating divergent transcriptomes (Supplemental Fig. S2A). Differential gene expression analysis (Supplemental Table S7) revealed 3982 genes that were upregulated and 4040 genes that were downregulated by NICD at E17.5 (Supplemental Fig. S2B). We performed gene ontology (GO) enrichment analysis separately for both upregulated and downregulated differentially expressed genes. GO terms that were enriched based on the significantly upregulated genes included “glial cell differentiation,” “gliogenesis,” and “OL development” (Fig. 5F). Conversely, downregulated GO terms included “positive regulation of neurogenesis,” “dendrite development,” and “regulation of neuron differentiation,” indicating suppression of neuron differentiation and maturation (Fig. 5G). In addition, many of the gliogenic transcription factors whose binding motifs were enriched within increased accessibility chromatin regions in ATAC-seq data from E16.5 also exhibited increased expression in RNA-seq data by E17.5, whereas expression of neurogenic transcription factors remained low (Supplemental Fig. S2C,D). Together, these results support that increased Notch signaling enhances gliogenesis at E17.5 while repressing neuron differentiation transcriptional programs.

Given the increased chromatin accessibility that we observed near SHH pathway genes at E16.5, we investigated whether increased Notch signaling influences SHH pathway gene expression in NICD progenitors at E17.5. GO enrichment analysis of upregulated genes highlighted “smoothened signaling pathway” as one of the most enriched terms (Fig. 5F), referring to the signal transduction process involving the SHH pathway component SMO (McCabe and Leahy 2015). Further examination of NICD differentially expressed genes revealed upregulation of *Smo* and SHH coreceptors *Boc* and *Gas1* (Fig. 5H). Additionally, NICD-overexpressing brains showed significant decreases in expression of known SHH pathway inhibitors, including *Hhip*, *Ptch2*, and *Ptch1* (Fig. 5H; Chen and Struhl 1996; Jeong and McMahon 2005; Holtz et al. 2013; Kwong et al. 2014; Zhulyn et al. 2015). *Gli1*, a downstream target of SHH signaling (Lee et al. 1997; Cohen et al. 2015), was decreased in NICD-overexpressing brains (Fig. 5H). We also identified upregulation of many cilia-associated genes, including *Iqce, Evc, Evc2*, and *Kif7* (Fig. 5H), which are known to positively regulate SHH signaling (Cheung et al. 2009; Liem et al. 2009; Allen et al. 2011; Izzi et al. 2011; Pusapati et al. 2014; McCabe and Leahy 2015). Interestingly, we observed that primary cilia of NICD-overexpressing progenitors appeared qualitatively shorter and more spherical than control cilia (Supplemental Fig. S2E), indicating possible regulation of ciliary structure by Notch signaling. Overall, our RNA-seq analysis at E17.5 demonstrated that increased Notch signaling prepares dorsal progenitors to respond to SHH by regulating components involved in SHH signal transduction at primary cilia. This suggests that Notch signaling primes these progenitors for increased sensitivity and responsiveness to SHH signaling.

We next wondered whether the transcriptional changes that we observed in response to Notch activation at E17.5 could be observed over the course of normal cortical development as progenitors switch from neurogenesis to gliogenesis. We therefore compared our RNA-seq data from E17.5 control neural progenitors with previously published RNA-seq data from E13.5 control neural progenitors (Supplemental Fig. S3A,B; Han et al. 2023). Differential expression analysis (Supplemental Table S8) revealed upregulation of *Notch1* and *Notch2* receptors at E17.5, along with increases in Notch ligands *Jag1* and *Jag2* (Supplemental Fig. S3B,C). Interestingly, Notch ligands *Dll1* and *Dll3*, along with some Notch target genes like *Hes1* and *Hey1*, were downregulated from E13.5 to E17.5 (Supplemental Fig. S3B,C). This may reflect differential usage of Notch ligands and effectors in Notch-mediated progenitor maintenance compared with Notch-mediated gliogenesis. In line with this idea, we observed a decrease in progenitor genes *Pax6* and *Sox2* at E17.5 (Supplemental Fig. S3B; Supplemental Table S8), along with increases in many genes involved in gliogenesis, including *Olig2*, *Ascl1*, *Egfr*, and *Sox10* (Supplemental Fig. S3C). In contrast, we observed decreases in several neurogenic genes, including *Neurog2*, *Neurod2*, *and Neurod6* (Supplemental Fig. S3C). These data indicate that during normal cortical development between E13.5 and E17.5, a shift in Notch signaling pathway components correlates with increased expression of genes involved in SHH signaling and gliogenesis, concomitant with decreased expression of genes involved in neurogenesis and progenitor maintenance.

**Figure 6.**
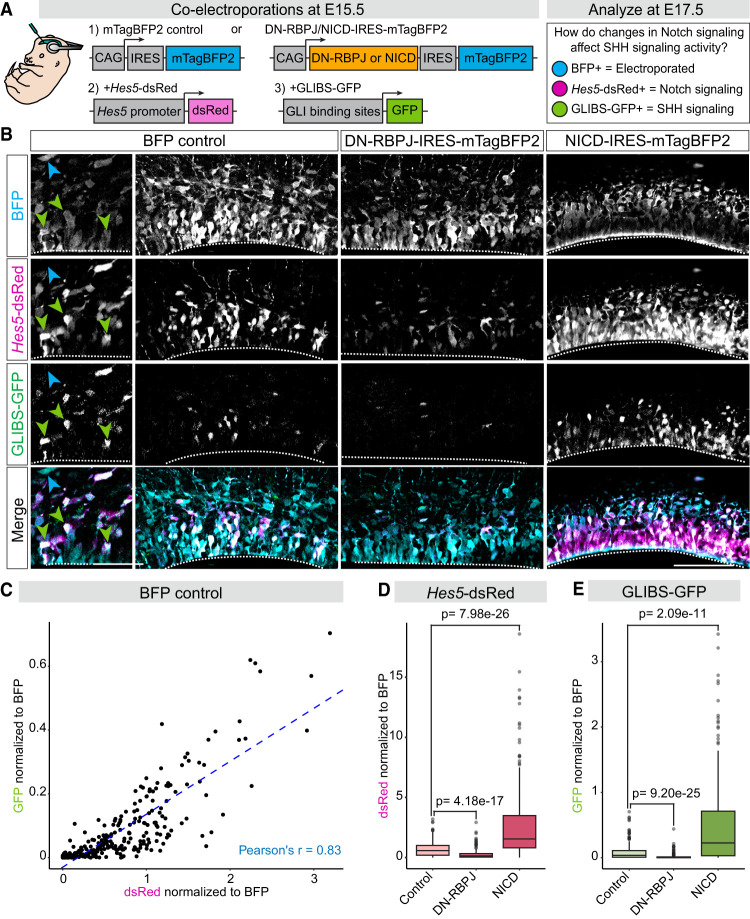
Notch signaling regulates SHH activity reporter levels in dorsal progenitors in vivo. (*A*) Schematic of constructs for BFP control, DN-RBPJ-IRES-mTagBFP2, NICD-IRES-mTagBFP2, *Hes5*-dsRed, and GLIBS-GFP. Wild-type mouse embryos were coelectroporated at E15.5 and analyzed for Notch and SHH reporter activity at E17.5. (*B*) Representative images of brains electroporated with BFP control, DN-RBPJ-IRES-mTagBFP2, or NICD-IRES-mTagBFP2 along with Notch (*Hes5*-dsRed) and SHH (GLIBS-GFP) activity reporters. The *left* panels in BFP control are zoomed-in images showing BFP^+^ cells that are *Hes5*-dsRed and GLIBS-GFP double positive (green arrows) and a BFP^+^ cell that is double negative for both reporters (blue arrow). Scale bar, 50 µm. (*Right*) Representative overview images of *Hes5*-dsRed and GLIBS-GFP fluorescence in the ventricular zone of BFP control, DN-RBPJ, and NICD brains. Dotted lines outline the ventral (*bottom*) limits of the pallium. Scale bar, 100 µm. Images for the GFP channel were adjusted with increased brightness for the purpose of visibility in the figure. (*C*) Graph of the normalized fluorescence intensity of dsRed plotted against the normalized fluorescence intensity of GFP, with each dot representing an individual cell in BFP control brains. The blue dotted line represents the line of best fit. The Pearson's *r* value represents the strength of linear correlation. (*D*,*E*) Quantification of the normalized fluorescence intensity of *Hes5*-dsRed (*D*) or GLIBS-GFP (*E*) in control, DN-RBPJ, and NICD (±SEM among biological replicates). Kruskal–Wallis test *P* < 2.2 × 10^−16^ for both *D* and *E*. Dunn's test was used to test significance between conditions, with *P*-values adjusted using the Bonferroni method. *N* = 267 BFP control cells, *N* = 285 DN-RBPJ cells, *N* = 290 NICD cells.

Given the influence of Notch signaling on the expression of SHH pathway components, we asked how manipulating Notch signaling up or down in neural progenitors would affect SHH signaling strength at E17.5. We used a GFP reporter under control of GLI binding sites (GLIBS-GFP) that has been used to measure SHH transcriptional activity (Hyman et al. 2009). To test whether Notch signaling affects SHH transcriptional activity in vivo, we coelectroporated GLIBS-GFP with DN-RBPJ-IRES-mTagBFP2, NICD-IRES-mTagBFP2, or BFP-only control at E15.5 and analyzed brains for reporter activity at E17.5 (Fig. 6A). We simultaneously monitored Notch signaling activity by coelectroporating a Notch transcriptional reporter under control of the *Hes5* promoter (*Hes5*-dsRed) (Mizutani et al. 2007). In BFP control brains, some electroporated BFP^+^ progenitors within the VZ exhibited signal from both the *Hes5*-dsRed and GLIBS-GFP reporters (Fig. 6B, green arrows), whereas others lacked signal from either reporter (Fig. 6B, blue arrow). Measuring the fluorescence intensity of the GLIBS-GFP and *Hes5*-dsRed reporters revealed that they were positively and linearly correlated in individual BFP^+^ progenitors, such that progenitors with higher *Hes5*-dsRed levels also had higher GLIBS-GFP levels (Fig. 6C). This correlation at the cellular level suggests that the coordination between Notch and SHH transcriptional activity occurs intrinsically within individual progenitors. As expected, DN-RBPJ significantly reduced the fluorescence levels of *Hes5*-dsRed, confirming DN-RBPJ's inhibitory effects on Notch signaling activity (Fig. 6B,D). We also observed that GLIBS-GFP signal was almost completely gone in DN-RBPJ electroporated brains (Fig. 6B,E), indicating that Notch pathway inhibition blocked SHH transcriptional activity in dorsal progenitors. Conversely, NICD overexpression increased the fluorescence intensity of both *Hes5*-dsRed and GLIBS-GFP (Fig. 6B,D,E). Therefore, Notch activation is sufficient to increase the levels of SHH transcriptional activity in dorsal progenitors. Furthermore, the observed changes in SHH transcriptional activity occurred within individual electroporated cells, supporting a cell-autonomous effect of Notch signaling on SHH pathway activity in the same cell. Altogether, these results indicate that Notch signaling regulates the response of progenitors to endogenous SHH signaling in the dorsal forebrain.

## Discussion

During embryogenesis, multipotent progenitor cells are directed toward distinct cell fates at specific developmental stages through a combination of intrinsic genetic programs and external signaling cues (Dorsky et al. 2000; Livesey and Cepko 2001; Guillemot 2007; Kohwi and Doe 2013). How do the intrinsic properties of progenitor cells shape their interpretation of external signals and the specific cell identities they adopt in response to those signals? To address this, we focused on the neuron–glia switch in the developing dorsal forebrain; a process in which neural progenitors transition from producing neurons to oligodendrocytes in response to a late embryonic SHH signal (Winkler et al. 2018). We previously identified Notch signaling as an important regulator of the timing of this switch (Tran et al. 2023), but the downstream mechanisms leading to the acquisition of oligodendrocyte fates remained unclear.

**Figure 7.**
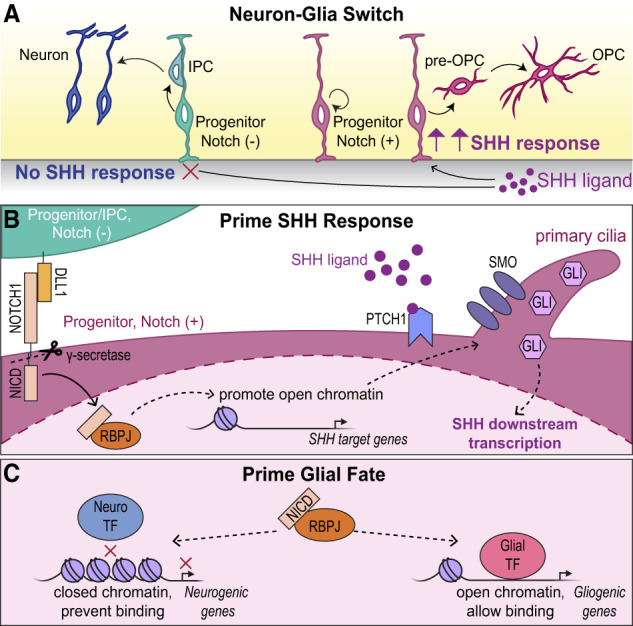
Proposed model for Notch signaling in regulating SHH response and glial fate specification. (*A*) During the neuron–glia switch, dorsal forebrain progenitors with low or no Notch activity do not respond to SHH ligand and differentiate into intermediate progenitor cells (IPC), which generate upper-layer excitatory neurons. Meanwhile, higher Notch signaling in other progenitors inhibits neurogenesis and maintains the progenitor state. These Notch (+) progenitors have increased sensitivity to the SHH ligand, which initiates production of pre-OPCs and OPCs. (*B*) Notch activity primes dorsal forebrain progenitors to respond to SHH. The Notch ligand DLL1 from Notch (−) cells binds to the NOTCH1 receptor of neighboring Notch (+) cells, leading to the cleavage of NICD by γ-secretase. NICD translocates to the nucleus and forms a transcriptional complex with RBPJ to activate transcription of unknown factors that open chromatin near SHH target genes, thereby enhancing SHH signaling and downstream transcription. (*C*) The NICD–RBPJ complex reduces chromatin accessibility near neurogenic genes and prevents binding to neurogenic transcription factors. NICD–RBPJ also promotes chromatin opening near gliogenic genes, allowing transcription factors to bind and promote glial fates.

Here, we now show that Notch signaling regulates the progenitor response to SHH, thereby facilitating the neuron–glia transition. We propose that Notch signaling establishes an internal progenitor state through three key mechanisms: (1) Increased Notch activity transcriptionally prolongs progenitor maintenance and prevents differentiation, (2) Notch signaling epigenetically and transcriptionally primes progenitors to respond efficiently to SHH, and (3) Notch signaling establishes a chromatin landscape that suppresses neuronal fates while priming progenitors for gliogenesis. These findings support a working model in which Notch signaling establishes progenitor sensitivity to SHH and competence for oligodendrocyte fates at both the epigenetic and transcriptional levels, permitting the timely production of oligodendrocytes when SHH signaling peaks in the dorsal forebrain (Fig. 7).

### Notch signaling regulates the response of dorsal forebrain progenitors to SHH

SHH signaling activity is initially low in the dorsal forebrain during early embryogenesis when neurogenesis is actively occurring. However, during later developmental stages, SHH signaling increases and triggers the shift to oligodendrocyte fate specification (Winkler et al. 2018). This rise in SHH signaling is facilitated by ligand secretion from migrating interneurons and the choroid plexus (Winkler et al. 2018). In our current study, ex vivo slice culture experiments revealed that Notch inhibition effectively blocked SHH-induced production of OLIG2^+^ cells, suggesting that Notch signaling is necessary for progenitors to respond to SHH and produce glia. Several studies have demonstrated significant cross-talk between the Notch and SHH pathways in various contexts, including the developing spinal cord and retina (Kong et al. 2015; Stasiulewicz et al. 2015; Ringuette et al. 2016; Ravanelli et al. 2018; Jacobs and Huang 2019, 2021). Notably, in the developing mouse and chick spinal cord, Notch activity regulates progenitor sensitivity to SHH. Consistent with this, we found that Notch overactivation in dorsal forebrain progenitors enhanced SHH signaling activity, whereas Notch inhibition nearly abolished it. Given that SHH ligand and downstream signaling components are expressed at lower levels in the dorsal forebrain compared with more ventral regions of the CNS (Dahmane et al. 2001; Tekki-Kessaris et al. 2001; Komada et al. 2008), Notch-dependent priming of progenitors for SHH responsiveness may be particularly important during the neuron–glia switch in the dorsal forebrain (Fig. 7A). This idea is further supported by our results showing that this requirement for Notch-dependent sensitization can be overcome by increasing SHH signaling downstream from the SHH ligand. However, cooperation of Notch and SHH during gliogenesis may be a more broadly conserved mechanism throughout the CNS and across species. Notch signaling is required for oligodendrocyte fate specification in the zebrafish spinal cord, where it maintains subsets of neural progenitors during neurogenesis and specifies them toward glial fates (Park and Appel 2003). Interestingly, constitutive Notch activity promotes excess OPCs in the zebrafish spinal cord but only at the place and time when OPCs normally form, suggesting that other signals like SHH are also required (Park and Appel 2003). In line with this idea, the Notch and SHH pathways have been shown to cooperate to drive oligodendrogenesis in the zebrafish spinal cord (Ravanelli et al. 2018).

Signaling cross-talk between Notch and SHH occurs at multiple levels, from transcriptional control to protein activity, and varies with biological context (Li et al. 2012; Kong et al. 2015; Stasiulewicz et al. 2015; Ringuette et al. 2016; Jacobs and Huang 2019, 2021). In spinal cord progenitors, Notch signaling modulates the localization of key SHH pathway components within primary cilia, a critical site for SHH signal transduction. Notch activation prevents PTCH1 from localizing to primary cilia, thereby allowing the accumulation of SMO and subsequent activation of GLI transcription factors (Kong et al. 2015; Stasiulewicz et al. 2015). Here, we provide evidence for an additional epigenetic regulatory mechanism in which Notch signaling modulates chromatin accessibility at SHH pathway genes in dorsal forebrain progenitors (Fig. 7B). Increased Notch signaling facilitated chromatin accessibility associated with genes encoding SHH pathway components. The presence of RBPJ binding motifs at chromatin peaks associated with SHH pathway genes suggests a potential direct regulatory role for Notch, consistent with a role for RBPJ in chromatin modulation through regulation of histone modifications in other contexts (Friedrich et al. 2022). Remarkably, expression of most SHH pathway components was initially unchanged at E16.5 but was more highly expressed in NICD-overexpressing progenitors 1 day later. Increased accessibility and higher expression of *Smo*, *Boc*, and *Gas1* and reduced expression of *Ptch1* and *Hhip* suggest that Notch activation may promote SHH pathway responsiveness through the upregulation of coreceptors while sustaining pathway activity by repressing inhibitors. A limitation of our bulk ATAC-seq and RNA-seq approaches is that they cannot resolve cell-autonomous versus non-cell-autonomous effects. However, our reporter assays demonstrated a strong correlation between Notch and SHH signaling within individual cells, suggesting that Notch signaling may modulate SHH transcriptional output in a cell-autonomous manner.

Interestingly, our reporter assays demonstrated increased GLI-dependent transcriptional output, even though *Gli1* mRNA expression was reduced in NICD progenitors and *Gli2* and *Gli3* expression levels remained unchanged. These results suggest that Notch signaling impacts SHH signaling upstream of GLI transcription factor expression. Because GLI proteins are expressed in the embryonic dorsal VZ/SVZ (Takanaga et al. 2009; Yu et al. 2009; Sousa and Fishell 2010; Wang et al. 2011) and can exist in full-length activator or cleaved repressor forms (Jacob and Briscoe 2003; Hui and Angers 2011; Sigafoos et al. 2021), it is possible that increased Notch activity influences their proteolytic processing. Consistent with this, Notch signaling can promote the full-length form of GLI3 in vitro (Stasiulewicz et al. 2015), raising the possibility that Notch may modulate the activator to repressor ratio of GLI proteins to fine-tune SHH responsiveness. One possible mechanism could be through expression of genes that modulate GLI processing within primary cilia. For example, we found that several ciliary genes were upregulated in NICD progenitors, including *Kif7*, *Iqce*, *Evc*, and *Evc2*, all of which are involved in GLI proteolytic processing in the cilium (Cheung et al. 2009; Liem et al. 2009; Dorn et al. 2012; Yang et al. 2012; Pusapati et al. 2014). Another possible mechanism could be through regulation of ciliary morphology or structure, given that Notch overactivation leads to elongation of floor plate cilia in the developing neural tube (Stasiulewicz et al. 2015). However, we observed the opposite in dorsal forebrain progenitors, where NICD brains exhibited shorter and wider primary cilia compared with controls. Although the presence of primary cilia is essential for SHH signaling in vertebrates (Sasai and Briscoe 2012; Bangs and Anderson 2017), the functional consequences of changing cilia length remain unclear (Macarelli et al. 2023). It is possible that shorter cilia may enhance signaling efficiency by increasing the local concentration of receptors and downstream effectors. For example, in fibroblasts, longer cilia increased the ciliary surface area, potentially diluting SHH pathway components and reducing signaling output (Breslin et al. 2014; Macarelli et al. 2023). Alternatively, Notch signaling may enhance SHH responsiveness not by elongating cilia but by regulating their structural integrity and molecular composition, such as the spatial localization of SHH signaling machinery within the cilium (Kong et al. 2015; Stasiulewicz et al. 2015). Further investigation into the distribution of SHH pathway components and post-translational regulation of GLI transcription factors will be necessary to clarify these mechanisms. Nonetheless, our transcriptomic analysis revealed upregulation of several positive regulators of SHH signaling, including ciliary components that control GLI post-translational processing, supporting the idea that Notch signaling acts on multiple parts of the pathway to activate and sustain SHH signaling (Fig. 7B).

### Notch-mediated epigenetic priming guides progenitor competence and cell fate decisions

Epigenetic priming is a process by which cells modify chromatin structure to make specific genes more accessible to regulatory factors, thereby facilitating gene expression and guiding cell fate transitions (Wang et al. 2015; McCarrey 2023). Our findings reveal that Notch signaling plays an important role in epigenetic priming of dorsal forebrain progenitors toward glial fates. ATAC-seq analysis of NICD-overexpressing progenitors showed increased chromatin accessibility at genes associated with progenitor, gliogenic, and neurogenic identities, suggesting a state of multilineage potential. In support of this, multilineage priming has been described in early embryonic dorsal progenitors, where expression of diverse neuronal subtype markers reflects a mixed transcriptional identity before these progenitors commit to specific fates (Li et al. 2020). Our RNA-seq analysis of NICD-overexpressing progenitors at E16.5 showed that progenitor-associated genes were highly expressed, whereas differentiation-specific mRNAs remained low. This result indicates an undifferentiated state in which these genes are epigenetically poised but not yet expressed.

Despite this multilineage potential, further ATAC-seq analysis revealed that the resulting chromatin landscape favored gliogenesis through two main mechanisms. First, increased Notch signaling promoted chromatin accessibility at gliogenic genes, including those associated with the oligodendrocyte lineage (Fig. 7C). Second, Notch activation reduced accessibility at regions containing neurogenic transcription factor binding sites while increasing accessibility at regions enriched with binding sites for gliogenic transcription factors (Fig. 7C). Notch signaling is well documented for its role in inhibiting neurogenesis by repressing the transcription of proneural factors (Ohtsuka et al. 1999; Nakamura et al. 2000; Kageyama et al. 2008), a pattern that we observed in our RNA-seq data. However, our findings suggest an additional mechanism by which Notch suppresses neurogenesis. By reducing chromatin accessibility at neurogenic regulatory sites, Notch activation may prevent transcription factor binding and activation of neurogenic programs. Conversely, increased accessibility for gliogenic transcription factor motifs suggests that epigenetic priming by Notch signaling predisposes progenitors toward glial fates (Fig. 7C). In fact, the gliogenic transcription factors whose binding motifs were enriched within more accessible chromatin regions also exhibited increased expression from E16.5 to E17.5, whereas neurogenic transcription factor expression remained low. Interestingly, NFI family binding motifs were identified within both increased and decreased accessibility regions, suggesting that Notch signaling may alter their binding patterns through chromatin remodeling. Given that NFI family transcription factors are implicated in both neurogenesis and gliogenesis (Mason et al. 2009), Notch-mediated changes in the chromatin landscape may direct NFI activity toward gliogenic rather than neurogenic transcriptional programs. This idea of Notch priming glial fates is further supported by our findings that by E17.5, NICD-overexpressing brains exhibited an enrichment of glial-related GO terms in RNA-seq data as well as increased production of OPCs. In the future, it will be interesting to determine what NICD–RBPJ targets directly modify chromatin to allow changes in accessibility and subsequently transcription.

In addition to its role in oligodendrogenesis, Notch signaling is also known to promote astrocytic differentiation (Kamakura et al. 2004; Namihira et al. 2009; Bansod et al. 2017; Gao et al. 2025). Thus, in addition to enhancing SHH signaling to promote oligodendrocyte lineage fates, the influence of Notch signaling on chromatin accessibility may be equally important for enabling progenitors to respond to astrocyte-inducing cues such as BMP and JAK–STAT signaling (Namihira et al. 2009; Katada et al. 2021). Further investigation will be essential to elucidate how Notch signaling differentially regulates oligodendrocyte versus astrocyte fate decisions.

### Changes in progenitor competence over developmental time

Previous studies have shown that progenitors undergo developmental changes over time, progressively maturing while maintaining a core progenitor identity defined by stable expression of progenitor markers. However, as they mature, their expression of other genes changes, which may alter progenitor competence to developmental cues at different times (Yang et al. 2014; Amadei et al. 2015; Moussy et al. 2017; Yuzwa et al. 2017). This progressive change in competence suggests that progenitors might also feature a flexible chromatin landscape that facilitates these changes while preserving their progenitor state. Supporting this idea, dorsal forebrain progenitors dynamically remodel their chromatin structure to determine whether they generate neurons or astrocytes in response to BMP signaling (Katada et al. 2021). Our results here uncover a role for the Notch pathway in this dynamic aspect of progenitor competence, facilitating chromatin modifications that enable progenitors to respond specifically to developmental cues as they arise. Thus, in addition to its established role in maintaining progenitor identity throughout brain development (Gaiano et al. 2000; Gaiano and Fishell 2002), Notch signaling also promotes gliogenic competence as development proceeds. These Notch-driven changes in chromatin accessibility appear to be important for determining how progenitors respond to SHH signaling at different developmental stages. For example, we demonstrate here that ectopic SHH activation at E15.5 induces progenitors to produce more glia and that this response requires intact Notch signaling and is enhanced by Notch pathway activation. Interestingly, the requirement for Notch signaling at E15.5 can be bypassed by overactivating SHH signaling cell-autonomously with SMOA1. However, a previous study found that cell-autonomous SHH activation in the early E12.5 cortex, when progenitors are normally neurogenic, did not promote gliogenesis but instead resulted in misspecified progenitors (Yabut et al. 2020). These results together suggest that progenitors at E12.5 are not yet competent to produce glia in response to SHH, whereas progenitors at E15.5 have gained this competence. The mechanisms that drive this temporal transition in Notch function, from supporting progenitor maintenance to promoting gliogenesis, may involve changes in downstream Notch effectors that favor gliogenic fates. In line with this idea, our transcriptomic analysis of control progenitors revealed a reduction in *Hes1* mRNA expression from E13.5 to E17.5 despite sustained expression of Notch receptors indicative of ongoing Notch activation. These results point toward a potential shift in Notch downstream signaling dynamics within progenitors over time. This finding aligns with our previous report demonstrating that HES1 maintains progenitors by repressing oligodendrogenesis (Tran et al. 2023), raising the possibility that *Hes1* downregulation may be a necessary step to permit oligodendrocyte fate specification through alternative NICD–RBPJ targets that may promote chromatin remodeling.

In summary, we propose that Notch signaling primes a chromatin landscape in dorsal progenitors that is both poised for gliogenic fates and responsive to SHH, ultimately promoting the specification and generation of glial lineages. This complex interplay underscores how Notch signaling orchestrates both immediate and future gene regulatory programs and positions progenitors to transition efficiently toward glial fates in response to SHH while also preserving the progenitor pool.

## Materials and methods

Details of reagents and tools used in this study are provided in Supplemental Table S9.

### Experimental model

#### Mouse lines

The following mouse lines were obtained from the Jackson Laboratory: B6 (C57BL/6J, stock no. 000664), R26-LSL-NICD [*Gt(ROSA)26Sor*^*tm1(Notch1)Dam*^/J (ROSA26^loxP-stop-loxP-Notch1-ICD^), stock no. 008159], and Emx1-Cre [B6.129S2-Emx1tm1(cre)Kri/J, stock no. 005628]. We also obtained timed pregnant Crl:CD1(ICR) mice (strain no. 022) from Charles River, which arrived at embryonic day (E) 12.5 and on which we performed electroporations at E15.5. For timed matings of B6 wild-type mice, we considered embryos to be at gestation day 0.5 on the day when the vaginal plug was detected. *Emx1-Cre (cre/cre)* mice were crossed with *R26-LSL-NICD (flox/flox)* mice to generate pregnant dams carrying double-heterozygous embryos for ATAC-seq, RNA-seq, and immunohistochemistry experiments at either E16.5 or E17.5. *Emx1-Cre* mice crossed with B6 mice were used as controls. Mouse lines were authenticated via genotyping by PCR. Animals were maintained according to the guidelines from the Institutional Animal Care and Use Committee of the University of Colorado Anschutz Medical Campus. The sex of the embryos was not determined for experiments.

### Method details

#### Expression plasmids

A *piggyBac* (PB) transposase system was used for in utero electroporation experiments to permit stable integration of the reporter plasmid into electroporated progenitors (García-Moreno et al. 2014). We found this approach to be necessary to label the oligodendrocyte lineage, in line with previous reports that episomal plasmids are silenced or lost in glial lineages (García-Marqués and López-Mascaraque 2013; Siddiqi et al. 2014).

DNA fragments for cDNA encoding a constitutively active version of mouse Smoothened (SMOA1) containing the point mutation W539L were synthesized and purchased from IDT as a gBlock. A SMOA1 cDNA gBlock was cloned using the NEBuilder HiFi DNA assembly master mix into a *piggyBac* (pPB) expression vector containing a CMV promoter, a CAG enhancer, and IRES-GFP (pPB-CAG-IRES-GFP). Generation of plasmids expressing a NOTCH1 intracellular domain (NICD; amino acids 1748–2293) and the dominant-negative version of RBPJ (DN-RBPJ) was described previously (Tran et al. 2023). The NICD and DN-RBPJ cDNA gBlocks were cloned into a pPB-CAG-IRES-mTagBFP2 backbone in which GFP was replaced by mTagBFP2. pPB-CAG-IRES-GFP and pPB-CAG-IRES-mTagBFP2 empty vectors were used as the GFP and BFP controls, respectively. All constructs were confirmed by restriction digest and whole-plasmid DNA sequencing (plasmidsaurus). The PBase expression plasmid CMV-mPB was described previously (Winkler et al. 2018).

For reporter assays, a plasmid expressing GFP under control of GLI binding sites (pGL3b-8xGliBS:EGFP or GLIBS-GFP) was used to analyze SHH signaling. pGL3b-8xGliBS:EGFP (Addgene plasmid 84602) was a gift from James Chen (Hyman et al. 2009). To analyze Notch signaling, we used a plasmid expressing dsRed under control of the *Hes5* promoter (*Hes5p*-DsRed or *Hes5*-dsRed). Hes5p-DsRed (Addgene plasmid 26868) was a gift from Nicholas Gaiano (Mizutani et al. 2007).

#### In utero electroporation

In utero electroporations were performed as described previously (Franco et al. 2011). Survival surgeries were performed on timed pregnant mice (E15.5), to expose their uterine horns. Approximately 1 μL of endotoxin-free plasmid DNA was injected into each embryo's lateral ventricles. Coelectroporations were performed with the following concentrations for each injection solution: SMOA1-IRES-GFP, DN-RBPJ-IRES-mTagBFP2, NICD-IRES-mTagBFP2, GFP control, BFP control, GliBS-GFP, or Hes5-dsRed at 0.75 mg/mL and CMV-mPB at 0.3 mg/mL. For E15.5 electroporations, five pulses of 100 msec, each separated by 950 msec, were applied at 45 V. Embryos were put back into the abdominal cavity, and the pregnant dams were sutured. Embryos were allowed to develop in utero for the indicated time before their brains were dissected.

#### Embryonic forebrain slice culture

Whole brains from E15.5 wild-type CD-1 or B6 mice or NICD mice were dissected and placed in ice-cold Complete HBSS (1× HBSS, 2.5 mM HEPES, 30 mM D-glucose, 1 mM CaCl_2_, 1 mM MgSO_4_, 4 mM NaHCO_3_). Brains were embedded in 3%–4% low-melting-point agarose dissolved in Complete HBSS and allowed to solidify on ice. Embedded brains were sliced using a vibratome (Leica VT1200S) into 300 µm thick slices and placed in Complete HBSS. Slices were transferred into uncoated Millicell cell culture membrane inserts in 6 well plates and cultured in slice culture media (Complete HBSS, basal medium Eagle, 20 mM D-glucose, 1 mM L-glutamine, penicillin–streptomycin) at 37°C, 5% CO_2_, and 100% humidity. For drug treatments, 100% DMSO was added to the slice culture media for a final concentration of 0.1% DMSO. One-hundred percent DMSO was used to make a 10 mM DAPT stock solution. One-hundred percent DMSO was also used to make a 100 µg/mL SHH ligand stock solution, which was added to the slice culture media for a final concentration of 100 ng/mL SHH and 0.1% DMSO. For SHH +DAPT treatments, SHH and DAPT stock solutions were added to slice culture media for a final concentration of 10 µM DAPT, 100 ng/mL SHH, and 0.1% DMSO. Slices were plated immediately with 1.5 mL of culture media containing DMSO, SHH only, or SHH + DAPT. After 1 day of incubation, 0.5 mL of culture media was removed and replaced with 0.5 mL of fresh media using the same drug concentrations. After 2 days in vitro (DIV), cell culture media were aspirated, and slices were washed in 1× PBS and fixed in cold 4% PFA for 30 min. Fixed slices were washed twice with 1× PBS and then used for immunohistochemical analysis as described below.

#### Immunohistochemistry

Whole embryonic brains were fixed in 4% paraformaldehyde (PFA) for 1 h at room temperature. Brains were sectioned coronally at 100 µm with a vibrating microtome. Sections from CTRL and NICD brains were histologically matched by aligning anatomical landmarks, such as the choroid plexus and ganglionic eminences, for comparison at approximately equivalent rostral–caudal regions. Free-floating sections were placed in 24 well plates and blocked with 500 μL of 10% donkey serum and 0.2% Triton X in 1× PBS for 2 h at room temperature. Blocking solution was then removed, and sections were incubated with primary antibodies in 10% donkey serum and 0.2% Triton X in 1× PBS overnight (16 h) at 4°C. Primary antibody solution was then removed, and sections were washed three times with 1× PBS for 5 min each at room temperature. After washing, sections were incubated with secondary antibodies in 1× PBS for 1 h at room temperature. Sections were then washed three times again using 1× PBS for 5 min each. Sections were mounted on glass slides with ProLong Diamond antifade mountant. Images were captured using a LSM900 Zeiss laser scanning confocal microscope in Airyscan 2 Multiplex 4Y mode at 20× or 40× magnification. Antibodies used for immunostaining are listed in Supplemental Table S9. The concentrations of the primary antibodies used were 1:1000 goat anti-OLIG2, 1:1000 rat anti-PDGFRA, 1:500 rabbit anti-TagRFP to detect mTagBFP2, 1:500 chicken anti-GFP, 1:500 rat anti-ARL13B, and 1:1000 mouse anti-PCNT. Donkey secondary antibodies conjugated to Alexa fluor 488, Rhodamine Red-X, Alexa fluor 647, or Alexa fluor 405 were used at 1:500.

#### Microdissection, tissue dissociation, and MACS

Whole brains from either E16.5 or E17.5 *Emx1-Cre(+/cre);R26-LSL-NICD(+/fl)* or *Emx1-Cre(+/cre)* mouse embryos were collected and microdissected for dorsal forebrain tissue in 1× neurobasal-A medium under a microscope. Microdissected tissue from two to five brains was pooled for each sample and then dissociated with the Worthington papain dissociation system according to the dissociation of mouse embryonic neural tissue protocol (document CG00053-Rev C) by 10x Genomics. Dissociated samples were strained with 30 µm MACS SmartStrainers into 1× HBSS and 10% FBS solution and centrifuged at 1000 rpm for 5 min to pellet the dissociated single cells. Neural progenitor cells were collected for each sample by magnetic-activated cell sorting (MACS) using anti-Prominin1 microbeads according to the manufacturer's recommended protocol.

#### RNA-seq and analysis

Following MACSorting of dorsal forebrain progenitors, RNA was extracted from each sample of —two to five pooled brains with the Zymo Quick-RNA micropreparation kit following the manufacturer's instructions. RNA concentration was measured with a QuBit RNA HS assay kit and submitted for cDNA library preparation and sequencing by the Genomics Shared Resource Facility at the University of Colorado Anschutz Medical Campus (RRID: SCR_021984). Libraries were prepared using a SMARTer stranded total RNA-seq kit for low-input ribo-depleted RNA and sequenced at 80 million reads/sample using the Illumina NovaSeq X platform. Three biological replicates each were obtained for the CTRL and NICD E16.5 and E17.5 conditions.

The nf-core RNA-seq pipeline (v 3.14.0) (Ewels et al. 2020) was used for data preprocessing and analysis with default parameters. STAR (Dobin et al. 2013) was specified in the nf-core pipeline for alignment to the GRCm38 mouse genome, and Salmon was specified for transcript quantification based on STAR alignments (Patro et al. 2017). Gene-level count tables resulting from Salmon were used as input to perform differential gene expression analysis using the DESeq2 R Bioconductor package (v 1.42.1) (Love et al. 2014). Genes were considered differentially expressed if found to have a Benjamini–Hochberg-adjusted *P*-value < 0.05. Principal component analysis plots and heat maps were generated using variance-stabilized transformed expression values obtained from DESeq2 and plotted using ggplot2 (v 3.4.1) (Wickham 2011) and the pheatmap package (v 1.012) (https://CRAN.R-project.org/package=pheatmap), respectively. GO term enrichment analysis was performed using the clusterProfiler package (v 4.10.1) (Yu et al. 2012).

For comparisons with RNA-seq data from E13.5 control neural progenitors, we obtained raw FASTQ files for control neural progenitors from the Dryad Digital Repository (dryad.B8RM0H) (Han et al. 2023). These raw reads were processed and analyzed alongside our E17.5 control RNA-seq data using the same pipeline described above.

#### ATAC-seq and analysis

Following MACSorting of dorsal forebrain progenitors, 50,000 cells from each sample were used to prepare an ATAC-seq library using the Zymo-Seq ATAC library kit following the manufacturer's instructions. Purified DNA library concentrations were measured using the Qubit DNA HS assay kit and submitted to the Genomics Shared Resource Facility at the University of Colorado Anschutz Medical Campus (RRID: SCR_021984) for sequencing with the Illumina NovaSeq X platform at 30 million reads/sample. Three biological replicates were obtained for CTRL and NICD E16.5 conditions.

The nf-core ATAC-seq pipeline (v 2.1.2) (Ewels et al. 2020) was used for data preprocessing and alignment to the GRCm38 mouse genome with default parameters. Reads mapped to the mitochondrial genome were removed. bam files resulting from BWA alignment (Li and Durbin 2009) in the nf-core pipeline were then filtered for fragments <100 bp in length using sambamba (v 0.8.2) (Tarasov et al. 2015) to isolate fragments likely originating from nucleosome-free regions (Buenrostro et al. 2013). Newly filtered bam files were then indexed using SAMtools (v 1.16.1) (Li et al. 2009) and used to generate bigwig files with deepTools (v 3.3.0) (Ramírez et al. 2016) for visualization of accessible chromatin peaks in Integrative Genomics Viewer (IGV) (Thorvaldsdóttir et al. 2013). Peaks were called using MACS2 (2.1.1.20160309) (Zhang et al. 2008) with the following parameters: -f BAMPE, ‐‐keep-dup all, -g mm, and ‐‐call-summits. Principal component analysis was performed using normalized read counts derived from consensus peaks with the DiffBind R Bioconductor package (v 3.12.0; https://bioconductor.org/packages/release/bioc/vignettes/DiffBind/inst/doc/DiffBind.pdf). TSS enrichment was visualized by computing signal profiles around transcription start sites using computeMatrix from deepTools, followed by plotting in ggplot2. Differential chromatin accessibility analysis was carried out using DiffBind and specifying DESeq2 for the analysis (https://bioconductor.org/packages/release/bioc/vignettes/DiffBind/inst/doc/DiffBind.pdf). Peaks with FDR < 0.05 were considered significant and differentially accessible. Significant peaks were annotated and assigned to the nearest TSS (−3000 bp and +3000 bp) using the ChIPseeker package (v 1.38.0) (Yu et al. 2015). Normalized read count plots were made by extracting the normalized read counts from the DiffBind object, filtering for significant peaks, and assigning peaks to the nearest TSS. Following annotation, peaks were categorized based on curated gene lists (progenitor genes, neurogenic genes, etc.), and the average normalized read counts across replicates were plotted using ggplot2. Normality of the data was tested using a Shapiro–Wilk's test, and variance was tested with a Levene's test. For comparisons where the data are not normally distributed, a Wilcoxon rank sum test was used to determine significance. To identify transcription factor binding motifs within differentially accessible sites, we used HOMER (v 4.9) (Heinz et al. 2010) to perform de novo motif enrichment analysis. To locate instances of motifs, we used HOMER's findMotifsGenome.pl -find, supplying position weight matrices from de novo motif enrichment results and the RBPJ position weight matrix from the HOMER motif database. For figures featuring genome tracks, bigwig files for each condition were merged using the SAMtools merge command to visualize averaged peaks.

### Quantification and statistical analysis

All images were captured using a LSM900 Zeiss laser scanning confocal microscope in Airyscan 2 Multiplex 4Y mode at 20× or 40× magnification. All images were exported in TIFF or JPEG format with no compression. Brightness, contrast, and background were adjusted equally for the entire image between controls and mutants using the “brightness/contrast” and “levels” function from the “image/adjustment” options in Adobe Photoshop or Fiji without any further modification.

For all immunostainings, three or more histological sections at three distinct rostral–caudal *z*-planes from each of —three to five different animals (at least nine sections total for each condition) were analyzed in the entire dorsal pallium. Biological replicates were individual animals. For slice cultures, biological replicates were individual slices from three to four different animals that were histologically matched at the rostral–caudal level between untreated and treated groups. Confocal, single-plane optical sections were used for quantification. Cells were analyzed in columns spanning the entire dorsal pallium across the lateral–medial axis. The total number of marker-positive cells was quantified from these columns in Fiji/ImageJ (Schneider et al. 2012) and then divided by the area of a column to get cell density (cells/mm^2^). The cell density was averaged across multiple columns and sections for each animal. For CTRL and NICD E17.5 brains, the cell density was quantified within the ventricular and subventricular zones. For electroporations, the percentage of electroporated cells that were positive for a marker was calculated and averaged for each animal. All statistical tests were performed using R. Normality of the data was tested using the Shapiro–Wilk's test, and variance was tested with the Levene's test. For independent two-group experiments, an unpaired two-tailed Student's *t*-test was used to determine statistical significance between two groups with equal variance. For comparisons between two groups with unequal variance, a Welch's *t*-test was used. For analysis involving three or more independent groups with equal variance, a one-way ANOVA was used followed by a Tukey's post hoc test. For analysis involving three or more independent groups with unequal variance, a Welch ANOVA test was used followed by a Games–Howell post hoc test. For analysis involving three or more independent groups that were not normally distributed, a Kruskal–Wallis test was used followed by a Dunn's post hoc test. Values were considered statistically significant at *P* < 0.05.

For reporter assay experiments, images were taken with the same settings across all sections from all conditions. Three distinct *z*-planes from two or more histological sections at matching rostral–caudal areas from each of three different animals were analyzed. Images were exported in TIFF format with no compression and with all three channels (RGB). For each image, a box 1000 pixels wide and 210 pixels high (∼100 µm) was drawn over the ventricular and subventricular zones in the dorsal pallium, with the ventricular wall at bottom of the box. Based on this box, the image was cropped and saved as a new TIFF image. Images in figures were linearly adjusted with increased brightness for the purpose of visibility in the figure. Analysis was performed on images without any adjustments. The fluorescence intensity of each image was analyzed using Fiji/ImageJ by drawing a region of interest (ROI) around every visible cell in the ventricular and subventricular zones in the cropped image; measuring the mean fluorescence intensity values for the red, green, and blue channels for each ROI; and saving the values in an Excel spreadsheet file. Values for the red and green channels were normalized to the corresponding values for the blue channel before statistical testing was performed using R. Biological replicates were individual cells circled as ROIs from three different animals. Normality was tested using the Shapiro–Wilk's test, and the equality of variances was tested using Levene's test. For data that did not meet the assumptions for one-way ANOVA, the Kruskal–Wallis test was used followed by the Dunn's post hoc test to determine significance between conditions. *P*-values were adjusted using the Bonferroni method. Values were considered statistically significant at *P* < 0.05. To determine the correlation of red and green values (normalized to blue), the Pearson's correlation coefficient was calculated.

### Data and code availability

All RNA sequencing and ATAC sequencing data are available through the Gene Expression Omnibus under accession series GSE295469, GSE295475, and GSE295476. All analysis code is described in the Materials and Methods and is available on GitHub at https://github.com/luulintran/notch_shh_manuscript.

## Supplemental Material

Supplement 1

Supplement 2

Supplement 3

Supplement 4

Supplement 5

Supplement 6

Supplement 7

Supplement 8
