# Supplementary material for: Epigenetic priming of neural progenitors by Notch enhances Sonic hedgehog signaling and establishes gliogenic competence
Source: Genes Dev. 2025 Jul 1;39(13-14):886–906. doi: 10.1101/gad.352555.124 (PMC12212003; doi:10.1101/gad.352555.124)
Supplement: Supplement 1 [file Supplemental_Data_050725.pdf]

SUPPLEMENTAL DATA

Figures

- Supplemental Fig. S1. The requirement for Notch signaling in oligodendrocyte lineage cell production is bypassed by increasing SHH signaling. Related to Figure 1.
- Supplemental Fig. S2. RNA-seq on NICD vs CTRL dorsal forebrain progenitors at E17.5. Related to Figure 5.
- Supplemental Fig. S3. Analysis of RNA-seq data from E13.5 and E17.5 control dorsal forebrain progenitors. Related to Figure 5.

Tables

- Supplemental Table S1. Excel file containing E16.5 RNA-seq differential gene expression data (NICD vs CTRL) too large to fit into a PDF. Related to Figure 2.
- Supplemental Table S2. E16.5 RNA-seq results of SHH pathway genes. Related to Figure 2.
- Supplemental Table S3. Excel file containing E16.5 ATAC-seq differential accessibility data (NICD vs CTRL) too large to fit into a PDF. Related to Figure 3.
- Supplemental Table S4. Excel file containing E16.5 ATAC-seq data related to neurogenic transcription factor binding motifs. Related to Figure 4.
- Supplemental Table S5. Excel file containing E16.5 ATAC-seq data related to gliogenic transcription factor binding motifs. Related to Figure 4.
- Supplemental Table S6. Excel file containing E16.5 ATAC-seq data related to the RBPJ binding motif. Related to Figure 4.
- Supplemental Table S7. Excel file containing E17.5 RNA-seq differential gene expression data (NICD vs CTRL) too large to fit into a PDF. Related to Figure 5.
- Supplemental Table S8. Excel file containing RNA-seq differential gene expression data (Control E17.5 vs E13.5) too large to fit into a PDF. Related to Figure 5.
- Supplemental Table S9. Reagents and tools table.

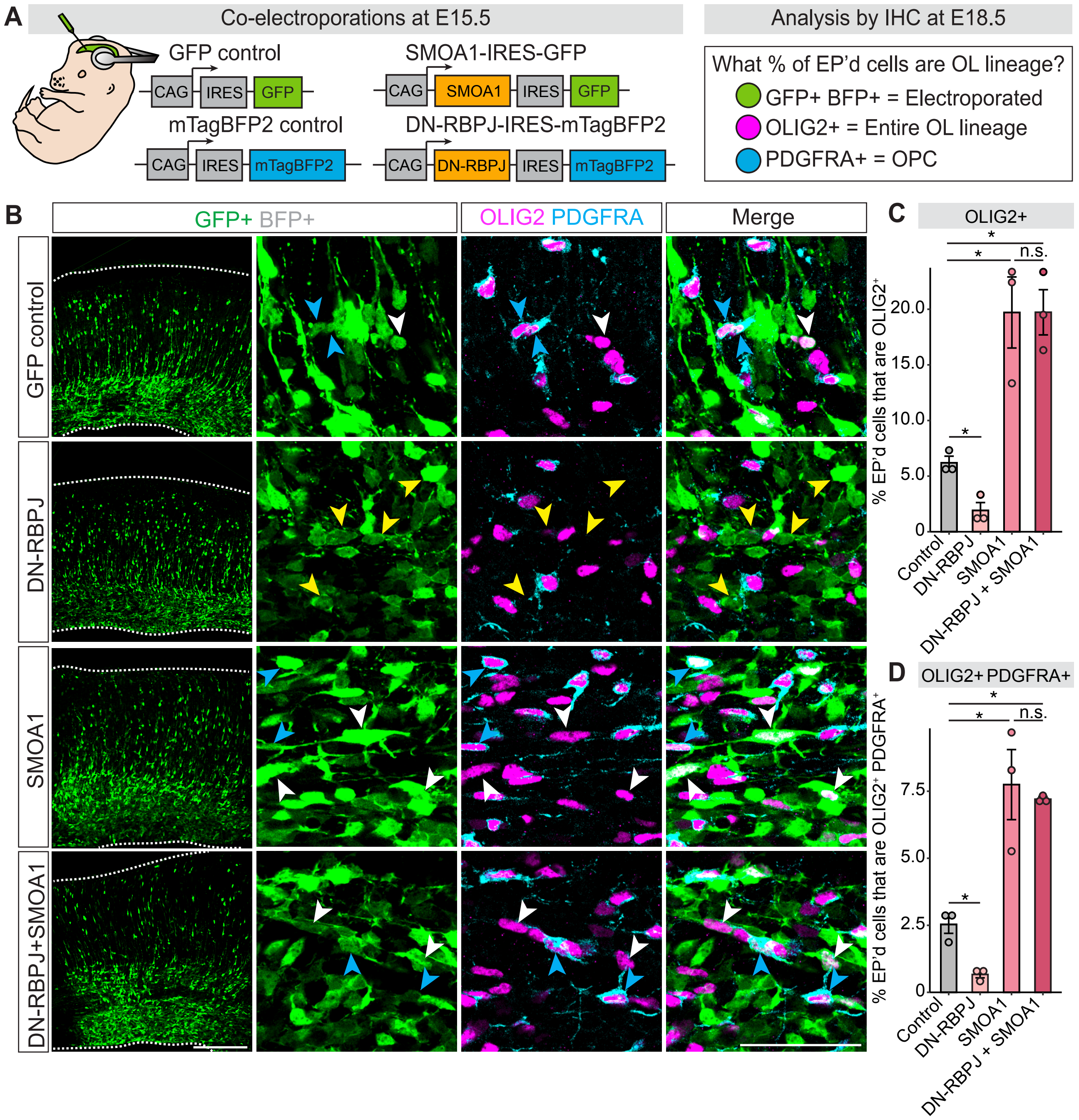

Supplemental Figure S1 (related to Figure 1). The requirement for Notch signaling in oligodendrocyte lineage cell production is bypassed by increasing SHH signaling. (A) Schematic of *in utero* electroporation approach. Wildtype mouse embryos were co-electroporated with GFP + BFP control, DN-RBPJ-IRES-mTagBFP2 + GFP, SMOA1-IRES-GFP + BFP, or DN-RBPJ + SMOA1 at E15.5. At E18.5, brains were dissected and analyzed by IHC for OLIG2 and PDGFRA to identify oligodendrocyte lineage cells. OL, oligodendrocyte; OPC, oligodendrocyte precursor cell. EP'd = electroporated. IHC = immunohistochemistry. (B) Left panels show overview images of the electroporations in the dorsal pallium. Since electroporated cells are both GFP+ and BFP+, the BFP channel is not shown. Dotted lines outline the dorsal (top) and ventral (bottom) limits of the pallium. Scale bar, 200 μm. Representative higher magnification images of brains electroporated with GFP control, DN-RBPJ, SMOA1, and SMOA1+DN-RBPJ. White arrowheads denote GFP+ OLIG2+ cells, blue arrowheads denote GFP+ OLIG2+ PDGFRA+ cells, and yellow arrowheads denote GFP+ OLIG2- PDGFRA- cells. Scale bar, 50 μm (C-D) Quantification of oligodendrocyte lineage cells among electroporated cells. Graphs show the average percentage (± SEM among biological replicates) of electroporated (GFP+ BFP+) cells that were OLIG2+ (C) or OLIG2+ PDGFRA+ (D). For comparisons between Control and DN-RBPJ, either Student's *t*-test for equal variance or Welch's *t*-test for unequal variance was performed. Welch's *t*-test Control vs DN-RBPJ: (C) $^*p= 0.009$; Student's *t*-test Control vs DN-RBPJ (D) $^*p= 0.0063$. For comparisons between Control, SMOA1, and DN-RBPJ+SMOA1, one-way ANOVA and Tukey's *post hoc* tests were performed. One-way ANOVA (C) $p = 0.0073$; (D) $p = 0.006$. Tukey's post-hoc test: $^*p< 0.05$, n.s. = not significant. N= 3 brains for each condition.

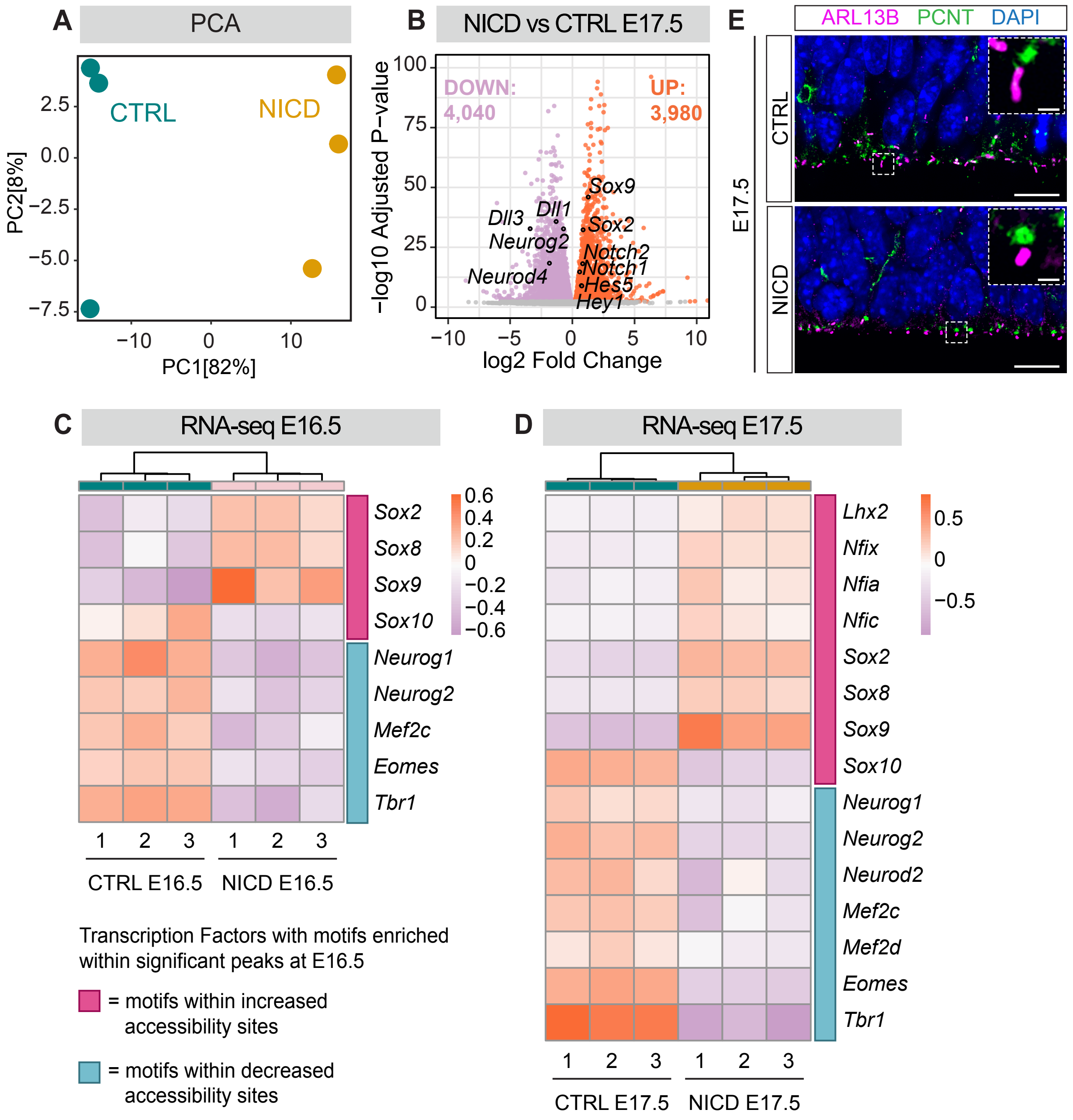

Supplemental Figure S2 (related to Figure 5). RNA-seq on NICD vs CTRL dorsal forebrain progenitors at E17.5. (A) PCA plot indicating separation of CTRL (N= 3) and NICD (N= 3) E17.5 transcriptomes by condition. PCA = Principal component analysis. (B) Volcano plot representing differentially expressed genes between CTRL and NICD. Differentially expressed genes with adjusted *p* value < 0.05 were considered to be significant. 4,040 genes were significantly downregulated (purple) and 3,980 genes were upregulated (orange) in NICD compared to CTRL. Selected Notch-related genes are outlined and labeled in the plot. (C-D) Heatmaps representing selected DEGs at E16.5 (C) or E17.5 (D) for transcription factors whose binding motifs were enriched in either increased or decreased accessibility sites found in E16.5 ATAC-seq analysis. Heatmap scales show variance-stabilized gene expression levels, with colors representing relative expression levels across samples. DEG = differentially expressed genes. (E) Representative images of the ventricular surface from E17.5 CTRL and NICD brains stained for ARL13B and PCNT. Overview images are shown on the left; scale bar, 10 μm. Insets show zoomed-in images of individual primary cilia; scale bar, 1 μm.

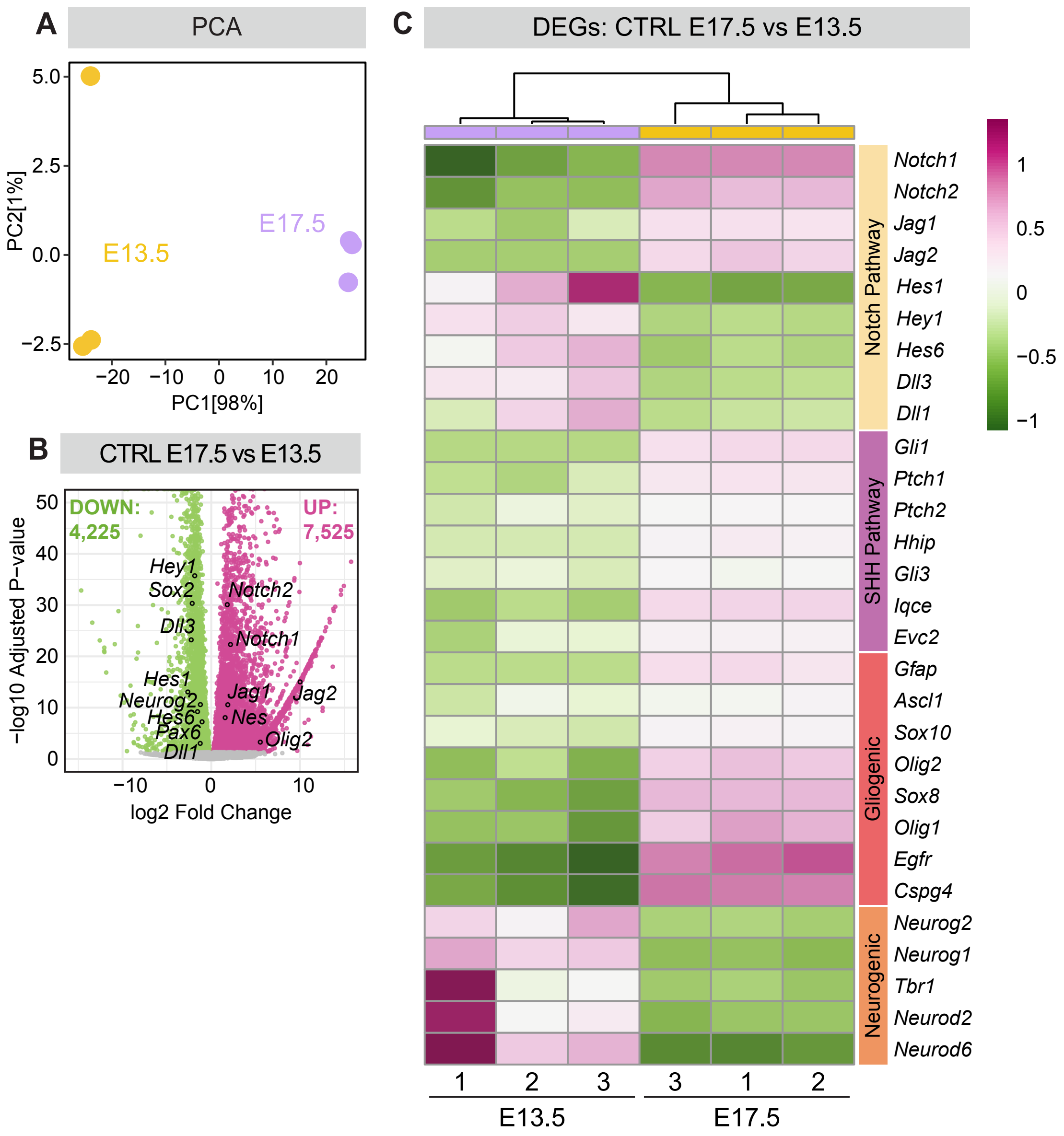

Supplemental Figure S3 (related to Figure 5). Analysis of RNA-seq data from E13.5 and E17.5 control dorsal forebrain progenitors. (A) PCA plot indicating separation of E13.5 (N =3) and E17.5 (N=3) transcriptomes by condition. PCA = principal component analysis. (B) Volcano plot representing differentially expressed genes between E13.5 and E17.5. Differentially expressed genes with adjusted p-value < 0.05 were considered to be significant. 4,225 genes were significantly downregulated (green) and 7,525 genes were upregulated (pink) at E17.5 compared to E13.5. Selected genes are outlined and labeled in the plot. (C) Heatmap representing selected differentially expressed genes related to the Notch pathway, SHH pathway, gliogenic cell identity, and neurogenic cell identity. Heatmap scale shows variance-stabilized gene expression levels, with colors representing relative expression levels across samples. DEG = differentially expressed genes.

Supplemental Table S2. E16.5 RNA-seq results of SHH pathway genes.

ensembl	symbol	entrez	baseMean	log2FoldChange	lfcSE	stat	pvalue	padj
ENSMUSG00000025407	Gli1	14632	581.078868	-1.8778382	0.16737024	-11.219667	3.26E-29	1.88E-26
ENSMUSG00000028681	Ptch2	19207	94.6735435	-2.7297505	0.41401621	-6.5933421	4.30E-11	3.70E-09
ENSMUSG00000064325	Hhip	15245	63.2238949	-1.1883386	0.43180108	-2.752051	0.00592233	0.05630482
ENSMUSG00000022687	Boc	117606	5761.54333	0.31415163	0.11931719	2.63291164	0.00846564	0.07222784
ENSMUSG00000021318	Gli3	14634	11172.1643	0.31288922	0.12787084	2.44691622	0.01440843	0.10451448
ENSMUSG00000021466	Ptch1	19206	1373.28588	-0.4604268	0.19052273	-2.41665	0.01566407	0.11051796
ENSMUSG00000038119	Cdon	57810	17770.6904	0.26583257	0.11009338	2.41461001	0.01575207	0.11092601
ENSMUSG00000073791	Efcab7	230500	132.879962	-0.5873952	0.27680881	-2.1220249	0.03383565	0.18498893
ENSMUSG00000001761	Smo	319757	3615.28976	0.23384972	0.1174593	1.99090005	0.04649188	0.22612738
ENSMUSG00000022812	Gsk3b	56637	13348.019	0.226223	0.11723617	1.92963491	0.05365209	0.24740702
ENSMUSG00000025231	Sufu	24069	3151.59319	-0.1910889	0.11960295	-1.5976942	0.11011105	0.38264263
ENSMUSG00000048402	Gli2	14633	4417.92676	0.15682448	0.10582652	1.48190146	0.13836652	0.43044689
ENSMUSG00000004364	Cul3	26554	4830.74045	0.13286624	0.0969723	1.37014631	0.17064123	0.47997396
ENSMUSG00000050382	Kif7	16576	2081.50887	0.1225576	0.11605069	1.05606957	0.29093643	0.62120497
ENSMUSG00000030768	Disp1	68897	581.726667	-0.1391591	0.17032877	-0.8170029	0.41392678	0.72566349
ENSMUSG00000052957	Gas1	14451	2892.16603	-0.0217583	0.19563264	-0.1112203	0.91144165	0.97441998
ENSMUSG00000036555	Iqce	74239	2635.17641	0.0041126	0.11389279	0.0361094	0.97119513	0.98973221

Supplemental Table S9. Reagents and tools table.

REAGENT or RESOURCE	SOURCE	IDENTIFIER
Antibodies		
Goat anti-OLIG2	R&D Systems	Cat# AF2418; RRID: AB_2157554
Rat anti-PDGFRA	Thermo Fisher Scientific	Cat# # 720219**;** RRID: AB_2633205
Rabbit anti-TagRFP	Thermo Fisher Scientific	Cat# R10367; RRID: AB_10563941
Chicken anti-GFP	Thermo Fisher Scientific	Cat# A10262; RRID: AB_2534023
Rat anti-ARL13B	BiCell Scientific	Cat # 90413; RRID: AB_3170226
Mouse anti-PCNT	BD Biosciences	Cat # 611814; RRID: AB_399294
Donkey secondary antibody anti-Chicken Alexa Fluor 488	Jackson ImmunoResearch	Cat# 703-545-155; RRID: AB_2340375
Donkey secondary antibody anti-Goat Alexa Fluor 647	Jackson ImmunoResearch	Cat# 705-605-147; RRID: AB_2340437
Donkey secondary antibody anti-Rabbit Alexa Fluor 405	Jackson ImmunoResearch	Cat# 711-475-152; RRID: AB_2340616
Donkey secondary antibody anti-Rat Rhodamine Red-X	Jackson ImmunoResearch	Cat# 712-297-003; RRID: AB_2340679
Donkey secondary antibody anti-Mouse Alexa Fluor 488	Jackson ImmunoResearch	Cat# 715-545-50; RRID: AB_2340846
DAPI	Thermo Fisher Scientific	Cat# D1306
Chemicals, peptides, and recombinant proteins		
DMSO	Millipore Sigma	Cat# D2650-100ML
DAPT	Millipore Sigma	Cat# 565770-5MG
SHH ligand (C25II), mouse	Genscript	Cat# Z03050
10x HBSS	Thermo Fisher Scientific	Cat# 14-185-052
1 M Hepes	Thermo Fisher Scientific	Cat# 15-630-106

1 M D-Glucose	Thermo Fisher Scientific	Cat# J60067
CaCl2	Millipore Sigma	Cat# C4901
MgSO4	Millipore Sigma	Cat# M2643-500G
NaHCO3	Millipore Sigma	Cat# S5761-500G
Basal Medium Eagle	Millipore Sigma	Cat# B1522-500ML
200 mM L-glutamine	Gemini Bio	Cat# 400-106
Penicillin-Streptomycin	Lonza Bioscience	Cat# DE17-602E
Millicell cell culture plate inserts	Millipore Sigma	Cat# Z353086
Cell culture 6-well plates	Greiner Bio-one	Cat# 657160
Neurobasal-A Medium (1X)	Gibco	Cat# 12349-015
Critical commercial assays		
NEBuilder HiFi DNA Assembly Master Mix	New England Biolabs	Cat# E2621S
ProLong Diamond Antifade Mountant	Thermo Fisher Scientific	Cat# P36961
Monarch MiniPrep Kit	New England Biolabs	Cat# T1010L
EndoFree Plasmid Maxi Kit	Qiagen	Cat# 12362
Papain Dissociation System	Worthington Biochem	Cat# LK003150
MACS SmartStrainers (30 μm)	Miltenyi Biotec	Cat# 130-098-458
Magnetic Separation columns	Miltenyi Biotec	Cat# 130-042-201
MACS MultiStand	Miltenyi Biotec	Cat# 130-042-303
Anti-Prominin-1 MicroBeads mouse	Miltenyi Biotec	Cat# 130-092-333
Zymo-seq ATAC library kit	Zymo	Cat# D5458
Quick-RNA Microprep kit	Zymo	Cat# R1050
Genomics Shared Resource	University of Colorado	RRID: SCR_021984
Deposited data		
Raw and processed E16.5 RNA-seq data	This paper	GEO accession #: GSE295475
Raw and processed E17.5 RNA-seq data	This paper	GEO accession #: GSE295476
Raw and processed E16.5 ATAC-seq data	This paper	GEO accession #: GSE295469
Experimental models: Organisms/strains		
B6 (C57BL/6J)	The Jackson Laboratory	stock no. 000664
R26-LSL-NICD (*Gt(ROSA)26Sor*$^{tm1(Notch1)Dam}$/J (ROSA26$^{loxP\text{-}stop\text{-}loxP\text{-}Notch1\text{-}ICD}$)	The Jackson Laboratory	stock no. 008159
Emx1-Cre (B6.129S2-Emx1tm1(cre)Kri/J)	The Jackson Laboratory	stock no. 005628
Crl:CD1(ICR**)**	Charles River	strain no. 022
Recombinant DNA		
CMV-mPB	Winkler et al., 2018	N/A
pPB-CAG-IRES-GFP	Tran et al., 2023	N/A

pPB-DN-RBPJ-IRES-mTagBFP2	Tran et al., 2023	N/A
pPB-NICD-IRES-mTagBFP2	This paper	N/A
pPB-SMOA1-IRES-GFP	This paper	N/A
pPB-CAG-IRES-mTagBFP2	This paper	N/A
pGL3b-8xGliBS:EGFP	Hyman et al., 2009	Cat# 84602; RRID: Addgene_84602
Hes5p-dsRed	Mizutani et al., 2007	Cat# 26868; RRID: Addgene_26868
Software and algorithms		
Fiji/ImageJ	Schneider et al., 2012	https://fiji.sc
Photoshop	Adobe	Adobe.com
Illustrator	Adobe	Adobe.com
R Studio	Posit	https://posit.co/download/rstudio-desktop/
Integrative Genomics Viewer (IGV)	IGV	https://igv.org/doc/desktop/
nf-core RNA-seq pipeline	Ewels et al., 2020	https://github.com/nf-core/rnaseq
nf-core ATAC-seq pipeline	Ewels et al., 2020	https://github.com/nf-core/atacseq
ggplot2	Wickham et al., 2011	https://github.com/tidyverse/ggplot2
DESeq2	Love et al., 2014	https://github.com/thelovelab/DESeq2
pheatmap	Kolde et al., 2015	https://github.com/raivokolde/pheatmap
clusterProfiler	Yu et al., 2012	https://github.com/YuLab-SMU/clusterProfiler
Sambamba	Tarasov et al., 2015	https://github.com/biod/sambamba
SAMtools	Li et al., 2009	https://github.com/samtools
Deeptools	Ramírez et al., 2016	https://github.com/deeptools/deepTools
MACS2	Zhang et al., 2008	https://github.com/macs3-project/MACS
Diffbind	Stark et al., 2011	https://github.com/hnthirima/DiffBind
ChIPseeker	Yu et al., 2015	https://github.com/YuLab-SMU/ChIPseeker

HOMER	Heinz et al., 2010	https://github.com/javrodriguez/HOMER
